# Cypher/ZASP drives cardiomyocyte maturation via actin-mediated MRTFA-SRF signalling

**DOI:** 10.7150/thno.98734

**Published:** 2024-07-22

**Authors:** Jialan Lyu, Zhicheng Pan, Ruobing Li, Hailong Yu, Yuesheng Zhang, Dongfei Wang, Xiang Yin, Yan He, Liding Zhao, Siyuan Chen, Shan Zhang, Hongqiang Cheng, Xiaogang Guo

**Affiliations:** 1 Department of Cardiology, The First Affiliated Hospital, Zhejiang University School of Medicine, Hangzhou, China.; 2 Department of Biochemistry, Zhejiang University School of Medicine, Hangzhou, China.; 3 Department of Pathology and Pathophysiology, and Department of Cardiology of Sir Run Run Shaw Hospital, Zhejiang University School of Medicine, Hangzhou, China.

**Keywords:** Cardiomyocyte maturation, Cypher/ZASP, SRF, Dilated cardiomyopathy

## Abstract

**Rationale:** Cardiomyocytes (CMs) undergo dramatic structural and functional changes in postnatal maturation; however, the regulatory mechanisms remain greatly unclear. Cypher/Z-band alternatively spliced PDZ-motif protein (ZASP) is an essential sarcomere component maintaining Z-disc stability. Deletion of mouse Cypher and mutation in human ZASP result in dilated cardiomyopathy (DCM). Whether Cypher/ZASP participates in CM maturation and thereby affects cardiac function has not been answered.

**Methods:** Immunofluorescence, transmission electron microscopy, real-time quantitative PCR, and Western blot were utilized to identify the role of Cypher in CM maturation. Subsequently, RNA sequencing and bioinformatics analysis predicted serum response factor (SRF) as the key regulator. Rescue experiments were conducted using adenovirus or adeno-associated viruses encoding SRF, both *in vitro* and *in vivo*. The molecular mechanisms were elucidated through G-actin/F-actin fractionation, nuclear-cytoplasmic extraction, actin disassembly assays, and co-sedimentation assays.

**Results:** Cypher deletion led to impaired sarcomere isoform switch and morphological abnormalities in mitochondria, transverse-tubules, and intercalated discs. RNA-sequencing analysis revealed significant dysregulation of crucial genes related to sarcomere assembly, mitochondrial metabolism, and electrophysiology in the absence of Cypher. Furthermore, SRF was predicted as key transcription factor mediating the transcriptional differences. Subsequent rescue experiments showed that SRF re-expression during the critical postnatal period effectively rectified CM maturation defects and notably improved cardiac function in Cypher-depleted mice. Mechanistically, Cypher deficiency resulted in the destabilization of F-actin and a notable increase in G-actin levels, thereby impeding the nuclear localisation of myocardin-related transcription factor A (MRTFA) and subsequently initiating SRF transcription.

**Conclusion:** Cypher/ZASP plays a crucial role in CM maturation through actin-mediated MRTFA-SRF signalling. The linkage between CM maturation abnormalities and the late-onset of DCM is suggested, providing further insights into the pathogenesis of DCM and potential treatment strategies.

## Introduction

Cardiomyocyte (CM) maturation is an essential stage in heart development that ensures potent contractions and efficient pumping throughout the lifespan of the organ 1, 2. During maturation, foetal CMs transform into adult CMs through adaptive structural and functional specialisations 1, 3. In particular, CMs exit the cell cycle, increase in size, adopt a rod-like shape, and undergo sarcomere assembly using mature gene isoforms to increase contractile force 4-6. Meanwhile, the sarcolemma invaginates and forms transverse (T)-tubules to enhance excitation-contraction coupling, and mitochondria expand to enable a metabolic switch from glycolysis to oxidative phosphorylation 7, 8. In addition, intercalated discs (ICDs) form between CMs to connect them tightly and transduce mechanical and electrical signals 1, 9. However, the regulatory mechanisms that underlie these effects remain greatly unknown.

Recent studies have established that sarcomeres play vital roles in CM maturation. Defective sarcomere assembly leads to aberrant T-tubule formation and mitochondrial oxidative metabolism 10, 11. Sarcomere mutations can also impair the genes or signalling pathways that coordinate CM maturation 10, 12, 13. Specifically, α-actinin-2, a critical sarcomeric protein to organize actin-filament alignment at Z-discs, drives cardiac structural and transcriptional maturation via serum response factor (SRF) signalling 10. Despite these studies, experimental evidence remains scarce.

The actin cytoskeleton is an essential component of sarcomeres and is present in two forms in CMs: filamentous (F)-actin and globular (G)-actin. Actin dynamics are known to regulate SRF signalling by modifying the nuclear translocation of its cofactor, myocardin-related transcription factor A (MRTFA) 14, 15. G-actin binds to MRTFA and sequesters it in the cytoplasm, while polymerisation into F-actin allows for the release and accumulation of MRTFA in the nucleus, thereby promoting SRF transcription 14-16. SRF has been reported to directly activate a series of key genes involving in sarcomere assembly, metabolism, and electrophysiology 17. Moreover, several sarcomeric proteins, such as cofilin-1, α-actinin-2, myotilin, and profilin, have the ability to regulate the assembly or disassembly of F-actin 10, 16, 18, 19. The potential role of actin-mediated MRTFA-SRF signalling as a connection between sarcomere and CM maturation is a topic that warrants further investigation.

Sarcomeric protein Cypher/Z-band alternatively spliced PDZ-motif protein (ZASP) is a member of PDZ-LIM protein family that is specifically expressed in cardiac and skeletal muscles 20, 21. It co-localises with α-actinin-2 to maintain the structural stability of Z-discs during contraction 21, 22. Cypher can be alternatively spliced: the long isoforms with three C-terminal LIM domains are known as Cypher long isoforms (CypherL), while the short isoforms without LIM domains are known as CypherS 23. Cypher/ZASP is closely related to dilated cardiomyopathy (DCM), characterised by obvious ventricular dilatation and systolic dysfunction 24. Global Cypher-null mice exhibit DCM and die shortly after birth 25. Cardiac-specific Cypher knockout during development or in adults causes DCM and premature death 26. In addition, mice with selective CypherL loss but not CypherS display late-onset DCM 27. Clinically, ZASP (human Cypher ortholog) mutations have been reported in multiple cardiac and skeletal myopathies 28-30, including DCM. Although Cypher/ZASP is important for normal cardiac function, whether Cypher/ZASP participates in CM maturation and thereby affecting heart function is unknown.

Here we found that Cypher deficiency perturbs CM maturation in both mice and neonatal rat CMs (NRCMs), which is mediated by actin-MRTFA-SRF signalling axis. SRF supplement during the critical postnatal period restores CM maturation and improves heart function in Cypher-depleted mice. Thus, Cypher/ZASP is a novel sarcomeric protein regulating CM maturation.

## Methods

### Mice

CypherL-KO mice with a C57BL/6 background were described previously 27. Heterozygous CypherL^+/-^ mice were crossed to generate CypherL-KO mice and wild-type littermate controls. Mice were euthanized by cervical dislocation under deep anaesthesia (3% isoflurane), followed by cardiac perfusion with phosphate-buffered saline. Heart tissues were collected from both male and female mice, the ages of which were indicated in the figure legends. Similar findings are reported for both sexes.

### AAV9 generation and administration

Adeno-associated virus serotype 9 encoding Flag-tagged SRF protein (AAV-SRF) and enhanced green fluorescent protein (AAV-EGFP) were purchased from Genechem (Shanghai, China). GV571 vectors harbouring the cardiac TnT promoter (cTNT:3Flag-SRF) were constructed to express SRF selectively in cardiomyocytes. Postnatal 1 day (P1) or 1 month (1M) mice were subcutaneously injected with AAV9 virus (1 × 10^10^ viral genomes [vg]/g).

### Echocardiography

Echocardiography was performed on adult mice using a Vevo 2100 Imaging System (Visual Sonics, Toronto, Canada) equipped with a 25 MHz transducer (model MS-400). Mice were anesthetised using isoflurane inhalation, with 3% isoflurane for induction and 1% for maintenance. M-mode and two-dimensional short-axis images were acquired at the papillary muscle level. Echocardiographic parameters were measured, including left ventricle internal dimension at systole (LVIDs) and left ventricular posterior wall thickness at systole (LVPWs). Functional parameters, including ejection fraction (EF), fractional shortening (FS), end-diastolic volume (EDV), and end-systolic volume (ESV), were calculated based on the primary parameters.

### NRCM culture

NRCMs were isolated from P1-P3 Sprague-Dawley rats using a Neonatal Heart Dissociation Kit (Miltenyi Biotech, Germany) according to the manufacturer's protocol. Briefly, CMs were separated and seeded on 10-cm dishes for 30 min at 37 °C to remove fibroblasts, plated on 1% gelatin pre-coated plates, and cultured in high glucose DMEM (Hyclone, USA) containing 10% foetal bovine serum (Gibco, USA), streptomycin (100 U/mL), and penicillin (100 U/mL). After incubation with BrdU (0.1 mmol/L) for 24 h to suppress fibroblast growth, NRCMs were transfected with small interfering RNA (siRNA) targeting Cypher (SiCypher) or scrambled siRNA (SiScramble) using Lipofectamine RNAiMAX (Invitrogen, USA). After 48 h, NRCMs were harvested for further experiments. SiCypher (target sequence: GGA ACA GCC UCU UCC ACA UTT) and SiScramble were purchased from GenePharma (Shanghai, China).

### Adenoviral infection of NRCMs

Adenoviruses carrying cDNA for rat SRF (Ad-SRF) and green fluorescent protein (Ad-GFP) were constructed by Genechem. When NRCMs reached 60-70% confluence, they were infected with Ad-SRF or Ad-GFP (MOI = 20). After 24 h, the adenovirus was removed and replaced with fresh medium with SiCypher or SiScramble for another 48 h, and NRCMs were harvested for further experiments.

### Immunofluorescence staining

Heart tissues were paraffin-embedded and cut into 4 μm sections that were dewaxed and rehydrated using an ethanol gradient. After antigen repair by microwave heating in ethylenediaminetetraacetic acid antigen retrieval buffer (Servicebio, China), sections were permeabilised using 0.2% Triton X-100 for 15 min, blocked with 5% bovine serum albumin for 1 h at room temperature (RT), and incubated with primary antibodies overnight at 4 ℃, followed by secondary antibodies for 2 h at RT. After sufficient washing with phosphate buffer saline-Tween, sections were counterstained with 4′,6-diamidino-2-phenylindole (DAPI) for 10 min to label nuclei and examined using a confocal microscope (Nikon A1).

Cells were fixed on coverslips with 4% paraformaldehyde for 10 min, washed with phosphate-buffered saline, and treated using the same methods as for tissue sections from the permeabilization step. The primary antibodies used for immunofluorescence are listed in Supplementary Table S2. The measurements of length and width were taken using the ImageJ straight-line tool. Colocalization index was assessed using Colocalization Image J plugin. The average fluorescence intensity was measured with background correction by Image J. To calculate the nuclear/cytosolic signal ratio, the freehand tool in Image J was used to select the nucleus and cytosol, respective fluorescence intensities were measured and the ratio was calculated afterwards.

### Transmission electron microscopy (TEM)

Heart tissues cut into small pieces were fixed with 2.5% glutaraldehyde for 4 h, post-fixed in 1% osmium tetroxide for 1 h, contrasted using 2% uranyl acetate for 30 min, and dehydrated using an ethanol gradient (50, 70, 90, 100%). Next, samples were infiltrated with acetone/Embed-812 resin mixtures (1:1 for 2 h, 1:3 overnight) at RT, embedded in Embed-812 resin and polymerised at 60 ℃ for 48 h. Ultrathin sections (70 nm thick) were cut using a Leica UC-7 microtome and stained with 2% uranyl acetate and lead citrate. Mitochondrion, myofibril, T-tubules and ICDs ultra structures were characterised using TEM (Titan G2 60-300, USA). A calibration ruler in each picture was used to convert pixels into distance within ImageJ software, then length, width, and area measurements were performed using straight-line and irregular area selection tools.

### Real-time quantitative polymerase chain reaction (RT-qPCR)

Total RNA was isolated from heart tissue or NRCMs using a Total RNA Mini-Prep Kit (Sangon Biotech, China) and reverse transcribed into cDNA using a HiScript®III qRT SuperMix (Vazyme, China). RT-qPCR was carried out using SYBR Green qPCR Master Mix (Vazyme) and the primer sequences listed in Supplementary Table S1. Relative mRNA expression was determined using the 2^-△△CT^ method and normalised to glyceraldehyde-3-phosphate dehydrogenase (GAPDH).

### Western blotting

Total protein was extracted from heart tissue or NRCMs using RIPA lysis buffer (Beyotime, China) supplemented with proteinase inhibitor (Sangon Biotech). Protein concentration was quantified using a bicinchoninic acid protein assay (Vazyme). After separation on 8 or 10% polyacrylamide gels, proteins were transferred onto polyvinylidene difluoride membranes (Millipore, USA), blocked with 5% bovine serum albumin for 1 h, incubated with diluted primary antibodies at 4 ℃ overnight, and probed with horseradish peroxidase-conjugated secondary antibodies at RT for 1 h. After washing, protein bands were visualised using enhanced chemiluminescence reagent (Millipore), and grey values were measured using Image J software. Relative protein levels were normalised to GAPDH or α-tubulin reference genes. The primary antibodies used for western blotting are listed in Supplementary Table S2.

### RNA-seq analysis

Total RNA was isolated from heart tissues collected from P3, 2M, and 6M WT and CypherL-KO mice (*n* = 3 per group), as well as from NRCMs treated with Cypher or scrambled siRNA (*n* = 3 per group). RNA was sequenced by LC Sciences (Hangzhou, China). Differentially expressed genes (DEGs) with a fold change of > 2 or < 0.5, and an adjusted *p* value < 0.05 were considered significant. Kyoto Encyclopedia of Genes and Genomes (KEGG) and Gene Ontology (GO) enrichment analysis were performed using the free cloud platform developed by LC Sciences (https://www.omicstudio.cn/). Gene set enrichment analysis (GSEA) was also performed on the cloud platform, and the top enriched pathways with normalised enrichment scores (NES) > 1 or < -1 and *p* < 0.05 were plotted. Heatmaps and volcano plots were generated using enhanced Heatmap v3.1 and Volcano v4.3.

### Prediction of transcription factors

Transcription factors were predicted based on the DEGs identified using RNA-seq analysis. The top 500 DEGs in P3 WT and CypherL-KO hearts, or in SiScramble and SiCypher treated NRCMs, were used for prediction in five different databases, including KnockTF (Knockdown/Knockout of Transcription Factors; http://www.licpathway.net/KnockTF/); BART (Binding Analysis for Regulation of Transcription; http://bartweb.org/); ChEA3 (ChIP-X Enrichment Analysis Version 3; https://maayanlab.cloud/chea3/); Lisa (epigenetic Landscape In Silico deletion Analysis; http://lisa.cistrome.org/); and TRRUST (Transcriptional Regulatory Relationships Unraveled by Sentence-based Text mining; https://www.grnpedia.org/trrust/). Intersecting elements from the five predictions were identified as putative transcription factors using a Venn diagram.

### G-actin and F-actin fractionation

The filamentous (F)-actin to globular (G)-actin ratio was examined using western blotting, as described previously 31. Firstly, hearts or NRCMs were collected, lysed with fresh lysis buffer (100 mM NaF, 50 mM KCl, 10 mM K_2_HPO_4_, 1 mM EGTA, 2 mM MgCl_2_, 1 M sucrose, 0.2 mM dithiothreitol, and 0.5% Triton X-100; pH 7.0) at RT for 30 min, and ultracentrifugation at 150,000 × *g* for 90 min at 24 ℃ using Optima MAX-XP Ultracentrifuge (Beckman, USA). Soluble actin (G-actin) in the supernatant was transferred to a new tube for detection. To isolate F-actin, pellets were re-suspended in an equal volume of F-actin depolymerisation buffer (20 mM Tris-HCl, 1.5 mM guanidine hydrochloride, 1 mM CaCl_2_, 1 mM sodium acetate, and 1 mM ATP; pH 7.5), and incubated on ice for 60 min with gentle shaking every 15 min. After F-actin had converted into soluble G-actin, the supernatant was centrifuged at 16,000 × *g* for 30 min and collected for detection. Equal amounts of the G-actin and F-actin fractions were analysed using western blotting.

### Co-immunoprecipitation (Co-IP)

Plasmids encoding Myc-tagged Cypher and Flag-tagged SRF or MRTFA were co-transfected into HEK293T cells using Lipofectamine 3000 (Invitrogen). The cell lysates were incubated with anti-Myc or anti-Flag primary antibodies overnight on a rolling shaker at 4 ℃, following by co-incubation with protein A/G magnetic beads (Bio-Rad) for 60 min. The beads were washed with PBS-T (PBS + 0.1% Tween 20) five times to reduce nonspecific binding. Then bound proteins were eluted and removed for western blotting. IgG was used as the negative control.

### Rho GTPase activity

Protein levels of Rho GTPases (Rhoa, Rac1, Cdc42) and their active GTP-bound forms were determined using the pull-down activation assay kit (NewEast Biosciences, China), according to the instructions. Briefly, the cell lysates were co-incubated with primary antibodies against active Rho GTPases and protein A/G agarose on a rolling shaker at 4 ℃ for 60 min. Then agarose was centrifuged at 5,000 × *g* for 1 min and washed three times, following by discarding the supernatant carefully and resuspending the pellet with assay buffer. Then pulled down proteins were subjected to western blotting. Rho GTPase activity was quantified by taking the ratio of active Rho to total Rho population.

### Latrunculin B washout assays

NRCMs were treated with 10 μM latrunculin B (Abcam, UK) for 1 h, rinsed three times with fresh medium, re-cultured for 2 or 4 h, fixed with 4% paraformaldehyde and processed as for regular staining. F-actin was labelled using Phalloidin-Alexa 488 (Abcam). Images were captured using a confocal microscope (Nikon A1).

### Cytoplasmic and nuclear protein extraction

Cytoplasmic and nuclear proteins were isolated from hearts and NRCMs using the NE-PER Nuclear and Cytoplasmic Extraction Reagents (Thermo, USA) according to the manufacturer's instructions. Briefly, 20 mg cardiac tissue or 2 × 10^6^ NRCMs were homogenised using 200 μL pre-cooled CER I, incubated on ice for 10 min with vigorous vortexing every 5 min, and centrifuged at 16,000 × *g* for 5 min at 4 °C. Supernatants were removed as the cytosolic fractions and pellets were dissolved in 100 μL pre-cooled NEB after washing with CER I. Resuspended pellets were incubated on ice for another 40 min with vortexing every 10 min, centrifuged at 16,000 × *g* for 5 min at 4 °C, and the supernatants were harvested as nuclear fractions. Total protein in the supernatants was quantified using bicinchoninic acid reagent (Vazyme) and analysed using western blotting with GAPDH as a cytosolic control and histone H3 as a nuclear control.

### Actin assembly and disassembly assays

*In vitro* actin assembly and disassembly assays were performed with the Actin Polymerization Biochem Kit (Cytoskeleton, USA) according to the manufacturer's instructions 19. The recombinant Cypher protein was synthesized and purified by Novoprotein Scientific Inc (Shanghai, China). For actin polymerisation assay, pyrene-labelled muscle actin was diluted with G-buffer in an ATP regenerating system and centrifuged at 14,000 × *g* for 30 min at 4 °C. Actin polymerization buffer (10 ×) was added to polymerize G-actin (2 μM) in the presence or absence of 2 μM purified Cypher. Pyrene fluorescence was recorded by SpectraMax i3x microplate reader (Molecular Devices, USA) in kinetic mode with an excitation wavelength of 365 nm and an emission wavelength of 407 nm every 30 s for 30 min. For actin depolymerisation assay, actin polymerization buffer (10 ×) was co-incubated with pyrene-labelled actin in G-buffer at RT for 1 h to preassemble actin filaments. Preformed actin filaments were diluted to 0.2 μM to induce actin disassembly in the presence or absence of 2 μM purified Cypher. Fluorescence was assayed using the same parameters every 30 s for 1 h. The initial fluorescence recorded at the beginning of the reaction was set as 100%.

### *In vitro* co-sedimentation assays

F-actin co-sedimentation assays were conducted using the Actin Binding Protein Spin-Down Biochem Kit (Cytoskeleton) 18. For F-actin binding assay, resuspended muscle actin in general actin buffer was co-incubated with actin polymerization buffer (10 ×) at RT for 1 h to preassemble actin filaments. The protein solution was pre-centrifuged at 150,000 × g for 1 h to remove large aggregates. Then purified Cypher, α-actinin, and BSA (final concentration 2-10 μM) were added to preformed F-actin (20 μM) for 30 min at RT respectively, followed by centrifugation at high speed (150,000 × g) for 90 min at RT. Supernatant and pellet fractions were separated on SDS-PAGE gel and stained with 0.1% Coomassie blue. Stained gels were imaged with the ChemiDoc MP imaging system (BioRad). F-actin binding proteins should pellet with the F-actin. α-actinin and BSA were recognized as the positive and negative control, respectively. For actin bundling assay, low speed centrifugation (14,000 × g) for 60 min was adopted to solely leave the bundled F-actin in the pellet after mixture incubation. The remaining steps were the same as above.

### Statistics

Data from at least three independent experiments were plotted as the mean ± SEM and analysed using GraphPad Prism version 9. Normal distribution and homogeneity of variance were tested using Kolmogorov-Smirnov and Bartlett's tests, respectively. Unpaired two-tailed Student's *t* tests were performed to compare two groups. One-way ANOVA with Tukey's post hoc test and two-way ANOVA followed by Bonferroni's post hoc test were used for multi-group comparisons. Differences between time-dependent curves were analysed using two-way repeated-measures ANOVA followed by Bonferroni's post hoc test. Statistically significant results were defined as *p* < 0.05.

## Results

### Impaired morphological maturation and sarcomere isoform switch in CypherL-KO hearts

**Figure 1:**
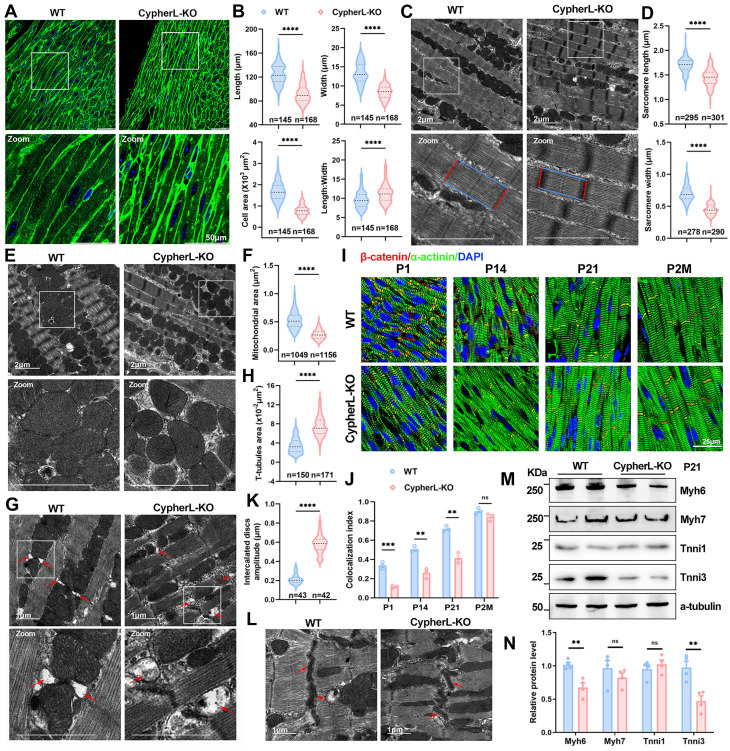
** CypherL loss impairs morphological cardiac maturation *in vivo*. (A)** Wheat germ agglutinin (WGA) staining of cardiac tissue sections from P21 wild-type (WT) and CypherL-knock-out (KO) mice. Boxes in upper panels are magnified in lower panels. Scale bars: 50 μm. **(B)** Length, width, length-to-width ratio, and cell area in cardiomyocytes (CMs) from P21 WT and CypherL-KO mice (*n* = 4). **(C-D)** Electron micrographs showing sarcomere structures in P21 WT and CypherL-KO hearts. Sarcomere length and width are highlighted using blue or red lines. Scale bars: 2 μm. (D) Statistical analysis (*n* = 4). **(E-F)** Electron micrographs of mitochondria from P21 WT and CypherL-KO hearts. Scale bars: 2 μm. (F) Mitochondrial area (*n* = 4). **(G-H)** Electron microscopy showing T-tubule dilatation (red arrows) in P21 CypherL-KO hearts. Scale bars: 1 μm. (H) Quantitative analysis (*n* = 4). **(I)** β-catenin distribution in intercalated discs (ICDs, yellow signal) from WT and CypherL-KO hearts at various developmental stages (from postnatal 1 day to 2 months of age). Scale bars: 25 μm. **(J)** Colocalization index of β-catenin with α-actinin was assessed using Colocalization Image J plugin and graphically represented (*n* = 3).** (K-L)** Electron micrographs of ICDs in P21 WT and CypherL-KO hearts. Scale bars: 1 μm. (K) Statistical analysis (*n* = 3). **(M-N)** Western blot analysis of immature (Myh7 and Tnni1) and mature (Myh6 and Tnni3) myofibrillar isoforms in WT and CypherL-KO hearts at P21, with α-tubulin as a loading control. (N) Quantitative analysis (*n* = 4). Data represent the mean ± SEM. Unpaired two-tailed Student's *t* test (B, D, F, H, K, J, N), **p < 0.01; ***p < 0.001; ****p < 0.0001; ns, no significant difference.

The critical perinatal period of cardiac maturation extends from embryonic day 18.5 (E18.5) to postnatal day 21 (P21) 32. Since Cypher is an essential cytoskeletal protein, we first examined its impact on CM morphology and ultrastructures. At P21, CypherL-KO CMs exhibited a significantly reduced cell area compared to WT cells due to a decrease in cell length, width, and diameter (Figure 1A, B and Supplementary Figure S1A, B). However, the length-to-width ratio increased (Figure 1B), indicating that CypherL loss had a greater effect on CM width. Then cardiac sections were imaged using transmission electron microscopy (TEM) to determine the alterations in ultrastructures. We found strikingly reduced sarcomere length and width (Figure 1C, D), smaller mitochondrial cross-sectional area (Figure 1E, F), and obvious T-tubule dilatation (red arrows, Figure 1G, H) in P21 CypherL-KO hearts. In addition, immunofluorescence showed that α-actinin retained a striated sarcomere pattern in CypherL-KO mice at P21, albeit with a decreased distance between Z-discs (Supplementary Figure S1C, D), which aligns with the observed reduction in sarcomere length as measured by TEM.

The intercalated discs (ICDs) are specialised CM junctions critical for mature cardiac tissue. They form during the maturation period and can be identified by the colocalization of β-catenin and α-actinin 33, 34. Notably, the absence of CypherL resulted in a diminished distribution of β-catenin at ICDs (yellow signal) in heart sections from P1 to P21 (Figure 1I, 1J), while exhibiting a comparable distribution in adult mice (2 months), suggesting a delayed maturation of ICDs. Additionally, more convoluted and wider ICDs were observed in P21 CypherL-KO hearts (Figure 1K, L), indicating the presence of structural abnormalities.

The transition from foetal to adult sarcomere isoforms is a well-established hallmark of myofibril assembly 1, 35. Therefore, we analysed the expression of immature (Myh7, Tnni1, Myl7, and Myl4) and mature (Myh6, Tnni3, Myl2, and Actc1) isoforms at different stages of cardiac maturation (E18.5-P21). RT-qPCR analysis demonstrated an obvious upregulation of immature isoforms such as Tnni1 and Myl7 in CypherL-KO hearts during the early perinatal stages (E18.5, P1.5; Supplementary Figure S1H), as well as the downregulation of mature isoforms including Myh6 and Tnni3 at the late postnatal stage (P7, P21; Supplementary Figure S1G). Analyses of the protein expression patterns of immature and mature myofibrillar isoforms in P3 and P21 CypherL-KO hearts yielded similar results (Supplementary Figure S1E, F and Figure 1M, N). Together, CM maturation in Cypher-depleted mice is abnormal as indicated by both morphological and molecular evidence.

### Aberrant myofibril maturation in Cypher-knockdown NRCMs

**Figure 2:**
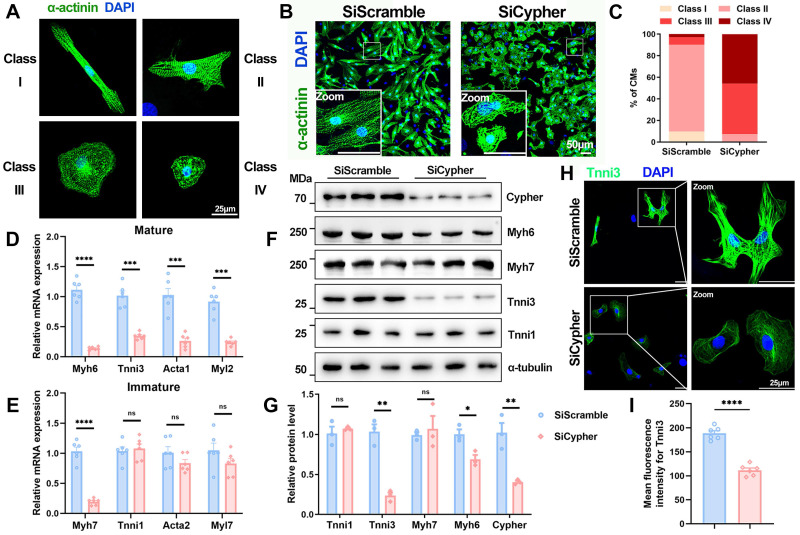
** Cypher is essential for myofibril maturation in neonatal rat cardiomyocytes (NRCMs). (A)** Representative images of typical class I-IV CM morphology. Blue, DAPI; green, α-actinin. Scale bars: 25 μm.** (B)** α-actinin immunostaining of NRCMs transfected with small interfering RNA against Cypher (SiCypher, *n* = 838 CMs) or scrambled small interfering RNA (SiScramble, *n* = 775 CMs). Scale bars: 50 μm. **(C)** Statistical analysis of CMs morphology grades. **(D-E)** Real-time quantitative polymerase chain reaction (RT-qPCR) analysis of mature (D) and immature (E) myofibrillar isoforms in NRCMs transfected with SiScramble or SiCypher (*n* = 6), with glyceraldehyde-3-phosphate dehydrogenase (GAPDH) as an internal reference. **(F-G)** Western blots of immature (Myh7 and Tnni1) and mature (Myh6 and Tnni3) myofibrillar isoforms in NRCMs treated with SiScramble or SiCypher, with α-tubulin as a loading control (*n* = 3). **(H-I)** Tnni3 staining of NRCMs transfected with SiScramble or SiCypher. Scale bars: 25 μm. (I) Quantitative analysis (*n* = 6), 6 randomly selected fields of view were quantified for each group in NRCMs. Data represent the mean ± SEM. Similar results were obtained from 3 independent cellular experiments. Unpaired two-tailed Student's *t* test (D, E, G, I), *p < 0.05; **p < 0.01; ***p < 0.001; ****p < 0.0001; ns, no significant difference.

Next, the role of Cypher in CM maturation was further explored in NRCMs, which can be classified into different maturation states (classes I-IV) *in vitro* according to cellular morphology and filament alignment^36^, ^37^ (Figure 2A). NRCMs transfected with control SiScramble mostly exhibited an elongated shape and well-aligned filaments (classes I and II), whereas those transfected with small interfering RNA against Cypher (SiCypher) displayed a round shape and disorganised filaments (classes III and IV; Figure 2B, C). In addition, sarcomere isoform switching was aberrant in Cypher-knockdown NRCMs, with significant decreases in the mRNA expression of mature isoforms (Myh6, Tnni3, Acta1, and Myl2; Figure 2D) but no changes in immature isoforms (Tnni1 and Acta2; Figure 2E). Western blot analysis also revealed that Cypher knockdown suppressed Myh6 and Tnni3 expression (Figure 2F, G), consistent with the RT-qPCR results. Finally, immunostaining showed clear Tnni3 downregulation in Cypher knockdown NRCMs (Figure 2H, I). Thus, Cypher appears to be critical for myofibril maturation in NRCMs.

### Impact of Cypher on transcriptional programmes involved in CM maturation

**Figure 3:**
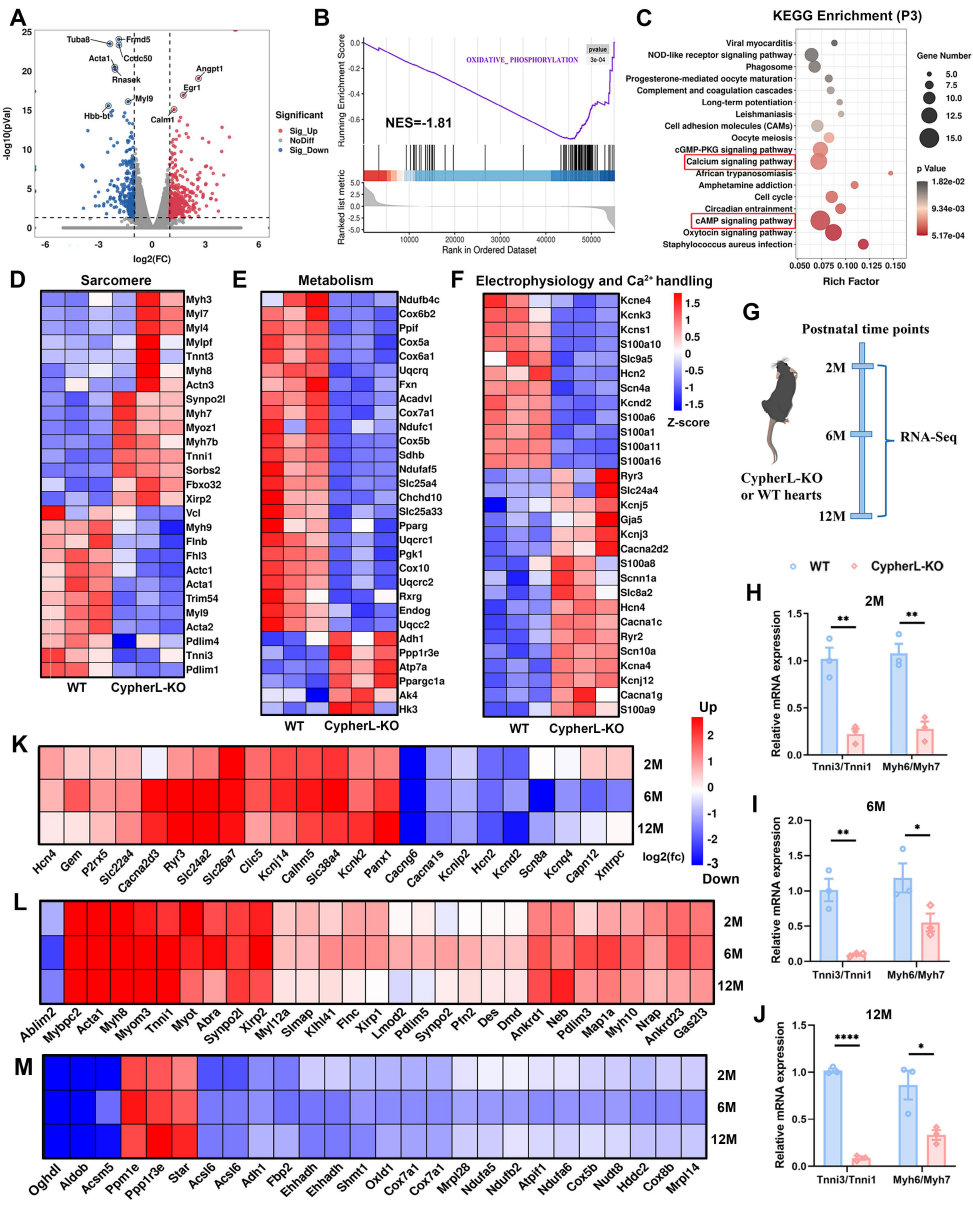
** Transcriptomic perturbation caused by CypherL ablation. (A)** Volcano plot showing differentially expressed genes (DEGs; *p* < 0.05, fold change > 2 or < 0.5) between WT and CypherL-KO mice (*n* = 3) at postnatal day 3 (P3). Upregulated genes are marked red, downregulated genes are marked blue. The top 10 genes are annotated. **(B)** Gene set enrichment analysis (GSEA) of the most significantly enriched pathway between WT and CypherL-KO mice at P3. **(C)** Kyoto Encyclopedia of Genes and Genomes (KEGG) analysis of the DEG in P3 CypherL-KO mice. Pathways with > 12 genes are highlighted by red boxes. **(D-F)** Heat map of major sarcomere (D), metabolism (E), electrophysiology and Ca^2+^ handing (F) genes in P3 WT and CypherL-KO mice. **(G)** RNA-seq analysis of hearts from WT and CypherL-KO mice (*n* = 3) were assessed at different time points (2, 6 and 12 months of age). **(H-J)** Tnni3/Tnni1 and Myh6/Myh7 reads per kilobase (RPKM) ratios in WT versus CypherL-KO hearts by RNA-seq at 2, 6, and 12 months (*n* = 3). Data represent the mean ± SEM. Unpaired two-tailed Student's *t* test was used, *p < 0.05; **p < 0.01; ****p < 0.0001. **(K-M)** The fold-changes of differential expression for major genes involved in electrophysiology and Ca^2+^ handing (K), sarcomere (L), and metabolism (M) in CypherL-KO mice at 2, 6, and 12 months of age.

To better elucidate the regulatory effects of Cypher on CM maturation at the transcriptomic level, we procured hearts from neonatal (P3) WT and CypherL-KO mice for RNA-seq. Differential expression analysis (*p* < 0.05, fold change > 2 or < 0.5) revealed that, in P3 CypherL-KO hearts, a total of 462 genes were up-regulated and 264 genes were down-regulated compared to WT hearts (Figure 3A). Gene set enrichment analysis (GSEA) demonstrated that oxidative phosphorylation was prominently enriched in P3 CypherL-KO hearts (Figure 3B). KEGG pathway analysis showed significant enrichment of cAMP and calcium signalling pathways in P3 CypherL-KO hearts (Figure 3C), which are implicated in the regulatory network of CM maturation 38.

Next, gene expression arrays revealed a decrease in the expression of mature sarcomere genes such as Myh9, Actc1, Acta1, and Tnni3, while an increase in the expression of immature genes, including Myl7, Myh7, Myh7b, and Tnni1 (Figure 3D), consistent with the observed sarcomere assembly defects in CypherL-KO hearts (Figure 1C, D). The expression of genes related to mitochondrial oxidative phosphorylation, including Ndufb4c, Cox6b2, and Cox5a, were observed to be down-regulated (Figure 3E), whereas glycolytic genes such as Ppp1r3ea and HK3 exhibited upregulation (Figure 3E), in line with the observed reduction in mitochondrial size following CypherL ablation (Figure 1E, F). Moreover, analysis of genes involved in mature electrophysiology and Ca^2+^ handling demonstrated a decrease in the expression of Kcnd2, Slc9a5, Scn4a, and Hcn2, alongside an elevation in the expression of Cacna2d2, Cacna1c, and Ryr2 in CypherL-KO hearts (Figure 3F). This observation aligns with the KEGG enrichment analysis of “calcium signalling” and T-tubule abnormality (Figure 3C and Figure 1G, H). Hcn4, an ion channel specifically expressed in immature CMs, was also up-regulated (Figure 3F). Altogether, RNA profiling suggests an important role of Cypher in regulating CM maturation from the transcriptome perspective.

### CM maturation defects in the progression of DCM caused by Cypher deletion

CypherL-KO mice display contractile defects from 6 months of age and then develop late-onset of DCM 27. Then we wonder how the maturation status varies during the development of DCM. Initially, the hearts of WT and CypherL-KO mice were subjected to RNA-seq analysis at different time points (2, 6, and 12 months, Figure 3G). It was observed that CypherL-KO hearts exhibited a significant decrease in the ratios of Myh6/Myh7 and Tnni3/Tnni1 at all three time points (Figure 3H-J). Additionally, genes critical for mature electrophysiology and Ca^2+^ handling (Figure 3K), sarcomere (Figure 3L), and mitochondrial metabolism (Figure 3M) were found to be dysregulated at these time points, including well-established markers such as Cacng6, Ryr3, Tnni1, Acsl6, and Cox5b.

Moreover, the ultrastructural characteristics of WT and CypherL-KO hearts were examined using TEM at the ages of 2, 6, and 12 months (Supplementary Figure S2). We found reduced sarcomere width (indicated by red lines), smaller mitochondrial cross-sectional area, obviously dilated T-tubules (indicated by red arrows) in the CypherL-KO hearts at 2, 6 and 12 months (Supplementary Figure S2), which was consistent with the findings observed at P21. While the change in sarcomere length (indicated by blue lines) exhibited an opposite pattern, with a decrease at 2 months (Supplementary Figure S2A, B) and an increase at 6 or 12 months (Supplementary Figure S2C-F). In addition, ICDs became progressively more convoluted and wider in CypherL-KO mice from P21 (0.58 ± 0.01 μm, Figure 1K, L) to 2 months (0.86 ± 0.03 μm, Supplementary Figure S2A, B), 6 months (1.46 ± 0.05 μm, Supplementary Figure S2C, E) and 12 months (1.49 ± 0.05 μm, Supplementary Figure S2D, F), aligning with the development of cardiac decompensation. These findings suggest a consistent presence of ultrastructural and transcriptional disorders associated with CM maturation throughout the progression of DCM induced by CypherL knockout.

### Serum response factor (SRF) is identified as the key downstream regulator

**Figure 4:**
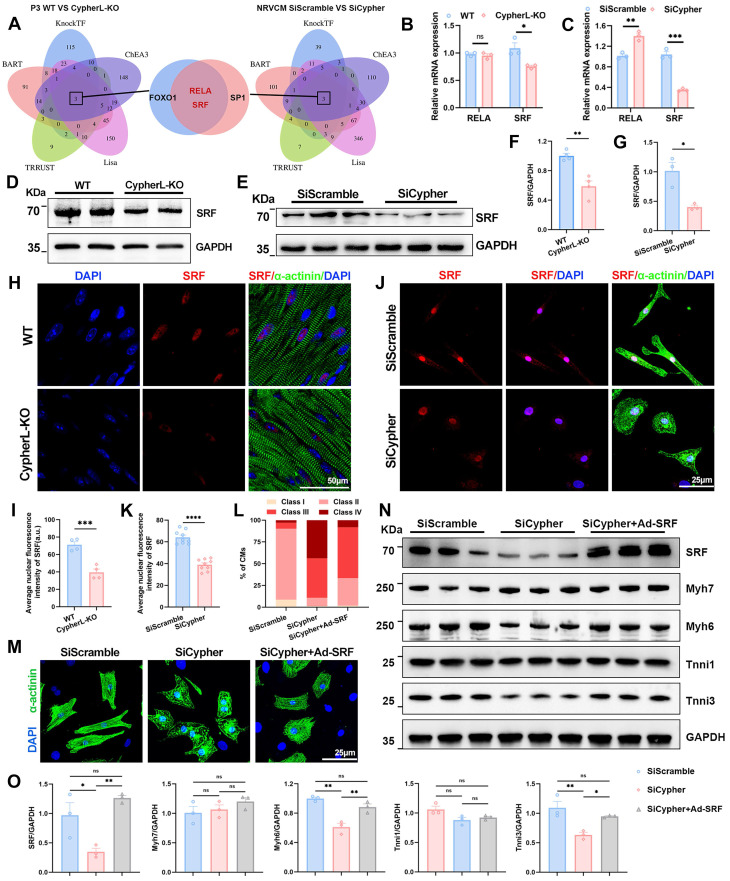
** SRF is the downstream regulator, and its re-expression rescues maturation defects in Cypher-knockdown NRCMs. (A)** Prediction of transcription factors based on the top 500 DEGs in P3 WT and CypherL-KO hearts (left panel) or in SiScramble- and SiCypher-treated NRCMs (right panel). Venn diagrams (middle panel) identifying RELA and SRF as putative transcription factors. **(B-C)** Relative mRNA expression of RELA and SRF in P3 WT and CypherL-KO hearts (B, *n* = 3), or in SiScramble- and SiCypher-treated NRCMs (C, *n* = 3). **(D-G)** Western blots of SRF in cardiac tissues from P3 WT and CypherL-KO mice (D and F, *n* = 3), or in SiScramble- and SiCypher-treated NRCMs (E and G, *n* = 3). GAPDH was probed as a loading control. **(H-K)** Immunostaining of SRF in cardiac tissue sections from P21 WT and CypherL-KO mice (H), or in control and Cypher-knockdown NRCMs (J). Nuclei and CMs were labelled with DAPI and α-actinin, respectively. Scale bars: 50 μm (H) or 25 μm (J). Average nuclear fluorescence intensity of SRF was quantified in mice (I, *n* = 4) or in NRCMs (K, *n* = 9), 9 randomly selected fields of view were quantified for each group in NRCMs. **(L-M)** α-Actinin immunostaining in NRCMs transfected with SiScramble (*n* = 1498 CMs), SiCypher (*n* = 1596 CMs), and SiCypher upon SRF-adenovirus (Ad-SRF) infection (*n* = 1485 CMs). NRCM morphology grades were quantified (L). Scale bars: 25 μm. **(N)** Western blots of SRF, immature (Myh7 and Tnni1), and mature (Myh6 and Tnni3) myofibrillar isoforms in NRCMs treated with SiScramble, SiCypher, and SiCypher upon Ad-SRF infection, with GAPDH as a loading control. **(O)** Quantitative analysis (*n* = 3). Data represent the mean ± SEM. Similar results were obtained from 3 (M, N) or 4 (E, J) independent cellular experiments. Unpaired two-tailed Student's *t* test (B, C, F, G, I, K) and one-way ANOVA with Tukey's post hoc test (O), *p < 0.05; **p < 0.01; ***p < 0.001; ****p < 0.0001; ns, no significant difference.

To figure out the key downstream regulator of Cypher in CM maturation, transcription factors were predicted based on the DEGs identified using RNA-seq. The top 500 DEGs between P3 WT and CypherL-KO hearts were selected for prediction using five online databases (BART, KnockTF, ChEA3, Lisa, and TRRUST). FOXO1, RELA, and SRF were predicted as putative transcription factors (Figure 4A, left panel). Using the same strategy, three transcription factors, SP1, RELA, and SRF, were identified using DEGs between control and Cypher knockdown NRCMs (Figure 4A, right panel). The Venn diagrams demonstrated the presence of RELA and SRF in two predictions (Figure 4A, middle panel). Subsequent analysis using RNA-seq (Figure 4B, C) and western blotting (Figure 4D-G) consistently showed the downregulation of SRF in P3 CypherL-KO hearts and Cypher knockdown NRCMs when compared with control. However, the expression patterns of RELA were inconsistent (Figure 4B, C), with no alterations in the former but an increase in the latter. Additionally, immunofluorescence staining exhibited a diminished nuclear distribution of SRF in P21 CypherL-KO mice (Figure 4H, I) or Cypher knock-down NRCMs (Figure 4J, K). Thus, the transcriptional changes observed in Cypher-deficient CMs are likely to be regulated by the transcription factor SRF.

### SRF re-expression rectifies maturation abnormalities in Cypher knockdown NRCMs

SRF, a transcription factor known to play a crucial role in CM maturation 10, 17, was investigated in this study as a potential downstream effector of Cypher in regulating CM maturation. To assess the impact of SRF on CM maturation, rescue experiments were conducted using adeno-associated virus encoding SRF in NRCMs. As expected, the re-expression of SRF partially rescued the maturation defects in NRCMs with Cypher knockdown, as evidenced by the presence of more well-arranged filaments (classes II and III; Figure 4L, M) and the reversal of expression patterns of mature isoforms (Myh6 and Tnni3) (Figure 4N, O). Additionally, the increased levels of SRF in control NRCMs did not have a significant effect on the expression of sarcomere isoforms (Supplementary Figure S3A, B). These results suggest that SRF re-expression can rectify CM maturation defects caused by Cypher knockdown.

### Cardiac SRF re-expression restores CM maturation and heart function in CypherL-KO mice

**Figure 5:**
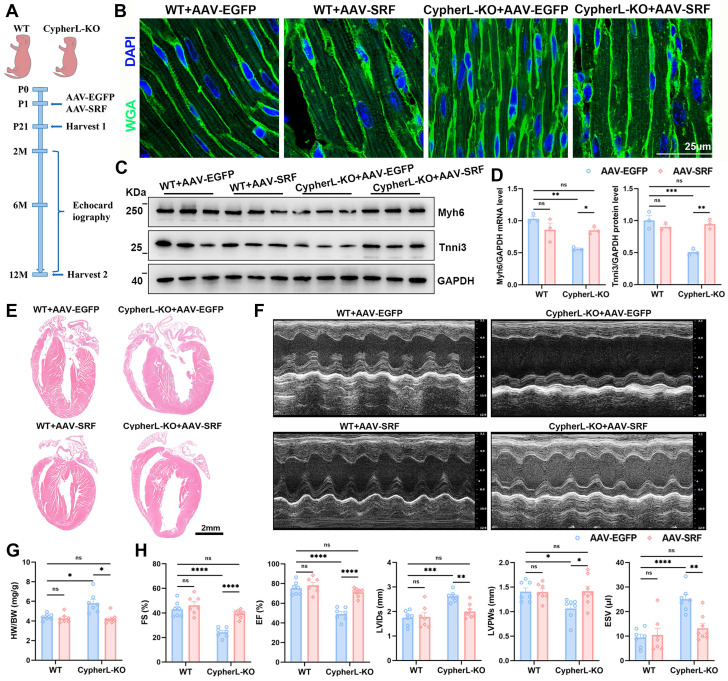
** SRF re-expression rectifies CM maturation and reinstates normal cardiac function in CypherL-KO mice. (A)** Schematic diagram of the experimental strategy. Neonatal (P1) WT and CypherL-KO mice were injected subcutaneously with a Cre-dependent adeno-associated virus encoding SRF (AAV-SRF) or control AAV-EGFP. A group of 21-day-old mice were utilized to evaluate alterations in CM maturation. Subsequently, additional mice were euthanized after echocardiography detection at the age of 2, 6, and 12 months. **(B)** WGA staining of cardiac tissue sections from P21 WT and CypherL-KO mice injected with AAV-SRF or control AAV-EGFP (*n* = 3). Scale bars: 25 μm. **(C-D)** Western blot analysis of mature (Myh6 and Tnni3) myofibrillar isoforms in P21 WT and CypherL-KO hearts infected with AAV-SRF or control AAV-EGFP, with GAPDH as a loading control (*n* = 3). **(E)** Representative H&E images of cardiac tissue sections from 12-month-old WT and CypherL-KO mice infected with AAV-SRF or control AAV-EGFP. Scale bars: 2 mm. **(F)** Representative echocardiographs of 12-month-old WT and CypherL-KO mice injected with AAV-SRF or control AAV-EGFP. **(G)** Heart weight to body weight (HW/BW) ratio (mg/g) of 12-month-old WT and CypherL-KO mice injected with AAV-SRF or control AAV-EGFP (*n* = 7-8). **(H)** Statistical analysis of FS, fractional shortening; EF, ejection fraction; LVIDs, left ventricle internal dimension at systole; LVPWs, left ventricular posterior wall thickness at systole; ESV, end-systolic volume. (*n* = 7-8). Data represent the mean ± SEM. Two-way ANOVA followed by Bonferroni's post hoc test (D, G, H), *p < 0.05; **p < 0.01; ***p < 0.001; ****p < 0.0001; ns, no significant difference.

Then rescue experiments were performed *in vivo* using AAV9-mediated cardiomyocyte-specific SRF expression (AAV-SRF; Figure 5A) on postnatal day 1. The protein levels of SRF at 21 days of age after AAV injection was shown (Supplementary Figure S3C-D). To assess the effect of SRF re-expression on CM maturation, an analysis was conducted on the cell area and sarcomere structures in the hearts of P21 mice. The reduced cell area (Figure 5B and Supplementary Figure S3G), CM diameter (Supplementary Figure S3E, F), and smaller sarcomere size (Supplementary Figure S3I-K), along with the decreased expression of mature sarcomere isoforms (Figure 5C, D) caused by CypherL ablation, returned to normal levels upon SRF re-expression. Additionally, WGA and actinin co-staining were performed to examine the number of sarcomeres within individual cardiomyocytes 39. The number of sarcomeres, which had significantly decreased due to CypherL deletion, nearly returned to WT control levels after SRF re-expression (Supplementary Figure S3L, M). Furthermore, the elevated expression of fetal cardiac genes (Nppa and Nppb) in P21 CypherL-KO mice was greatly diminished following SRF re-expression (Supplementary Figure S3H).

Next, we examined whether SRF re-expression can improve heart function in adult CypherL-KO mice. Echocardiographic assessment of cardiac function revealed no significant differences between WT and CypherL-KO mice at 2 months of age (Supplementary Figure S3N). However, at 6 and 12 months of age, CypherL-KO mice developed DCM, as evidenced by significant ventricular dilatation and systolic dysfunction compared to WT mice (Supplementary Figure S3O and Figure 5E-H). Re-expression of SRF significantly ameliorated this DCM phenotype, as indicated by improved ejection fraction (EF) and fractional shortening (FS), along with decreased left ventricular internal diameter in systole (LVIDs) and end-systolic volume (ESV) (Supplementary Figure S3O and Figure 5E-H). Notably, the cardiac function of CypherL-KO mice with SRF re-expression exhibited a nearly equivalent level to that of WT controls at both 6 and 12 months of age (Supplementary Figure S3O and Figure 5E-H). Heart to body weight ratio (HW/BW) was strikingly increased in CypherL-KO mice and returned to normal levels when SRF was re-expressed (Figure 5G).

To further investigate the relationship between defective CM maturation and the development of DCM, rescue experiments were performed using AAV-SRF at postnatal 1 month (Supplementary Figure S4A), a time that surpasses the critical window for CM maturation. The protein expression of SRF was shown (Supplementary Figure S4B, C). It was observed that CypherL-KO mice with AAV-SRF injection at 1 month still exhibited severe DCM at the age of 6 months, as evidenced by significant ventricular dilatation and systolic dysfunction (Supplementary Figure S4D-F). The cardiac function of these mice closely resembled that of CypherL-KO mice (Supplementary Figure S4F). Taken together, SRF re-expression during the critical postnatal period restores CM maturation and improves heart function in CypherL-KO mice.

### Cypher promotes SRF signalling via actin-mediated MRTFA nuclear localisation

**Figure 6:**
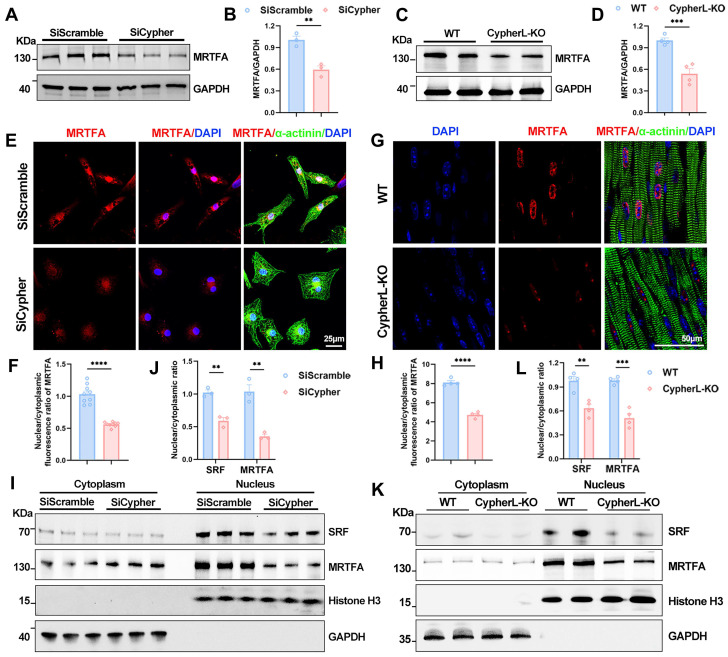
** Cypher promotes SRF signalling by regulating MRTFA nuclear localisation. (A-B)** Western blot analysis of total MRTFA in SiScramble- and SiCypher-treated NRCMs. GAPDH was probed as a loading control. (B) Statistical analysis (*n* = 3).** (C-D)** Western blot of total MRTFA in P21 WT and CypherL-KO hearts (*n* = 4). GAPDH was used as the internal reference.** (E-F)** Immunostaining of MRTFA in control and Cypher-knockdown NRCMs labelled with DAPI (nuclei) and α-actinin (CMs). Scale bars: 25 μm. Nuclear to cytoplasmic fluorescence ratio of MRTFA, 9 randomly selected fields of view were quantified for each group (F).** (G-H)** Representative fluorescent images of MRTFA in cardiac tissue sections from P21 WT and CypherL-KO mice. Nuclei and CMs were labelled with DAPI and α-actinin, respectively. Scale bars: 50 μm. Nuclear to cytoplasmic fluorescence ratio of MRTFA was quantified (*n* = 4).** (I-L)** Western blots of cytosolic and nuclear SRF or MRTFA in NRCMs transfected with SiScramble and SiCypher (I), or in P21 WT and CypherL-KO hearts (K). GAPDH and Histone H3 were used as cytosolic and nuclear markers, respectively. Quantification of nuclear/cytoplasmic ratio of SRF and MRTFA in NRCMs (J, *n* = 3) or in mice (L,* n* = 4). Data represent the mean ± SEM. Similar results were obtained from 3 independent cellular experiments (A, E, I). Unpaired two-tailed Student's *t* test (B, D, F, H, J, L), **p < 0.01; ***p < 0.001; ****p < 0.0001.

Subsequently, we investigated the mechanism by which Cypher regulates SRF signalling during CM maturation. SRF transcription is known to be activated by its cofactor, MRTFA 14, 15. Firstly, the western blot analysis showed an obvious reduction in protein levels of MRTFA in both Cypher knock-down NRCMs and P21 CypherL-KO mice (Figure 6A-D). Then immunostaining was performed to figure out the subcellular localisation. Cypher knock-down dramatically inhibited MRTFA nuclear accumulation in NRCMs, as evidenced by the reduced nuclear/cytoplasmic fluorescence ratio of MRTFA (Figure 6E, F). Similar results were observed in P21 CypherL-KO heart sections (Figure 6G, H). Further analysis of nucleocytoplasmic assays revealed that SRF and MRTFA predominantly localized within the nuclear fractions, with their nuclear/cytoplasmic ratios significantly decreasing following Cypher knockdown or knockout (Figure 6I-L). In addition, we observed a similar expression pattern for SRF and MRTFA. In Cypher knock-down NRCMs, the protein levels of SRF and MRTFA were markedly reduced (Supplementary Figure S5A, B). After intervention with an adeno-associated virus encoding SRF, SRF expression significantly increased, accompanied by a rise in MRTFA protein levels (Supplementary Figure S5A, B). However, co-immunoprecipitation experiments demonstrated no interaction between Cypher and either SRF or MRTFA (Supplementary Figure S5C, D).

**Figure 7:**
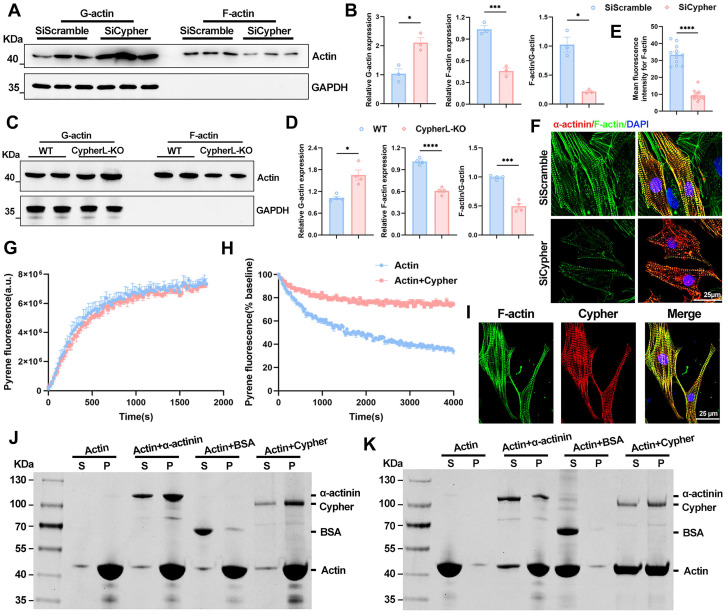
** Cypher regulates actin dynamics by binding and stabilizing F-actin. (A-D)** Western blots of globular actin (G-actin) and filamentous actin (F-actin) in SiScramble- and SiCypher-treated NRCMs (A) or in P21 WT and CypherL-KO hearts (C), with GAPDH as a loading control. G-actin and F-actin were quantitatively analyzed in NRCMs (B, *n* = 3) or mouse myocardium (D,* n* = 4). **(E-F)** Immunofluorescence analysis of F-actin in Cypher-deficient NRCMs labelled with phalloidin (F-actin), DAPI (nuclei) and α-actinin (CMs). Scale bars: 25 μm. (E) F-actin fluorescence intensity, 12 randomly selected fields of view were quantified for each group.** (G-H)** Effects of Cypher on the kinetics of actin polymerisation (G) and depolymerisation (H) were assessed using pyrene-labelled actin. 3 independent experiments were repeated.** (G)** Actin polymerization buffer was added to polymerize G-actin (2 μM) in the presence or absence of 2 μM Cypher. Fluorescence was assayed every 30 s for 30 min. **(H)** Preformed pyrene-labelled actin filaments were diluted to 0.2 μM to induce actin filament disassembly in the presence or absence of 2 μM Cypher. Fluorescence was assayed every 30 s for 1 h. **(I)** Co-localisation of F-actin and Cypher in NRCMs. Scale bars: 25 μm.** (J-K)** F-actin binding or bundling assay. Cypher was incubated with F-actin and then centrifuged at high speed for 90 min (J, 150,000 × g) or low speed for 60 min (K, 14,000 × g). Supernatant (S) and pellet (P) fractions were separated on SDS-PAGE gel and stained with 0.1% Coomassie blue. α-actinin: positive control; BSA: negative control. Data represent the mean ± SEM. Similar results were obtained from 3 independent cell experiments (A, F, G, H, J, K). Unpaired two-tailed Student's *t* test (B, D, and E), *p < 0.05; ***p < 0.001; ****p < 0.0001.

How does Cypher modulate MRTFA-SRF signalling? Actin dynamics is closely associated with the nuclear localisation of MRTFA 14, 15. To assess the levels of G/F-actin, an actin pelleting assay was conducted, wherein F-actin was pelleted through ultracentrifugation, leaving G-actin in the supernatant 10, 31. The results revealed a significant decrease in the F-actin to G-actin ratio in the absence of Cypher, attributed to a decrease in F-actin and an increase in G-actin in both mice and NRCMs (Figure 7A-D). This reduction in F-actin was further confirmed through phalloidin immunostaining in NRCMs (Figure 7E, F). Rho GTPase activity functions as the upstream regulator of actin-MRTFA signalling 40. The protein levels of active GTP-bound forms of Rho GTPases (Rhoa, Rac1, Cdc42) were evaluated, and no significant alterations in Rho GTPases activity were observed in Cypher knock-down NRCMs (Supplementary Figure S5E, F). Thus, it can be inferred that Cypher modulates actin dynamics to regulate MRTFA-SRF signalling.

To better clarify the relationship between Cypher-actin-MRTFA signalling and CM maturation, NRCMs were treated with latrunculin B (actin polymerisation inhibitor, 0.1 μM) and CCG-1423 (MRTFA inhibitor, 10 μM) for 24 h 41. In comparison to the control group, the administration of latrunculin B and CCG-1423 led to a reduction in the expression of SRF, MRTFA and mature sarcomere isoforms (Myh6 and Tnni3; Supplementary Figure S6A, B), as well as hindered the maturation of NRCMs, which had a round morphology and disorganised filaments (classes III and IV; Supplementary Figure S6C, E). Meanwhile, the nuclear accumulation of SRF was significantly diminished by the administration of latrunculin B or CCG-1423 (Supplementary Figure S6C, F). In addition, latrunculin B and CCG-1423 inhibited the nuclear localisation of MRTFA (Supplementary Figure S6D, G) and yielded a comparable phenotype (classes III and IV) and expression of proteins associated with maturation (Myh6, Tnni3, and SRF) as observed in Cypher knockdown NRCMs (Supplementary Figure S6A-G). These findings suggest that Cypher regulates CM maturation through actin-MRTFA-SRF signalling.

### Cypher modulates actin dynamics by binding and stabilizing F-actin

To further explore the role of Cypher in actin dynamics, latrunculin B (10 μM) washout experiments were performed in NRCMs 42. Latrunculin B treatment for 1 h rapidly disrupted actin filaments in control and Cypher knock-down NRCMs (Supplementary Figure S7A). The stress fibres in control NRCMs exhibited rapid re-assembly 2 h after latrunculin B washout, and largely returned to their normal state 4 h after drug washout (Supplementary Figure S7A, B). However, the stress fibres in Cypher knock-down NRCMs were unable to recover and remained predominantly disturbed filaments after latrunculin B washout (Supplementary Figure S7A, B), indicating that Cypher has a significant impact on actin dynamics. In control NRCMs, the nuclear translocation of MRTFA was observed concomitantly with the reassembly of actin filaments (Supplementary Figure S7C, D), whereas the nuclear accumulation of MRTFA remained at a low level before and after drug washout in Cypher knock-down NRCMs (Supplementary Figure S7C, D), in line with disturbed actin dynamics.

Next, we aimed to investigate the potential influence of Cypher on the processes of actin polymerisation and depolymerisation. The kinetics of actin polymerisation or depolymerisation were evaluated by employing pyrene-labelled actin 19. Specifically, actin polymerization buffer was added to polymerize G-actin in the presence or absence of purified Cypher. The fluorescence measurement results showed that the addition of Cypher did not exhibit any discernible impact on the kinetics of actin polymerisation (Figure 7G). Subsequently, preformed pyrene-labelled actin filaments were subjected to a 10-fold dilution to induce actin filament disassembly, with or without the addition of purified Cypher. The presence of Cypher significantly inhibited the decrease in fluorescence rate (Figure 7H), indicating that Cypher may act to stabilize F-actin filaments.

Finally, we wondered how Cypher modulates F-actin depolymerisation. Co-localisation analysis revealed that Cypher strongly co-localised with F-actin in NRCMs (Figure 7I). To assess the F-actin binding or bundling activity of Cypher, F-actin co-sedimentation assay was performed. Purified Cypher was co-incubated with preformed F-actin and then centrifuged at high speed (Figure 7J, 150,000 × g) or low speed (Figure 7K, 14,000 × g), the former leaves all the F-actin in the pellet, whereas the latter leaves only the bundled F-actin 18. The protein α-actinin, which is known to bind or bundle F-actin, was observed to co-sedimented with F-actin and served as the positive control (Figure 7J, K). The analysis of F-actin binding (Figure 7J, 150,000 × g) showed that purified Cypher was mostly detected in the pellet fraction, similar to α-actinin, suggesting its role as a F-actin binding protein. Meanwhile, upon centrifuging at low speed (Figure 7K, 14,000 × g), F-actin was found in the supernatant in the absence of Cypher, but in the pellet (bundled F-actin) in the presence of Cypher, indicating that Cypher promotes the assembly of F-actin bundles.

Together, Cypher binds and stabilizes actin filaments. It modulates actin dynamics and enhances MRTFA nuclear localisation, thereby promoting SRF signalling during CM maturation.

## Discussion

Cypher/ZASP is recognized as a sarcomeric protein that is anchored at Z-disc 21, 43. It is not deemed necessary for the process of sarcomerogenesis or Z-disc formation, but it does play a crucial role in maintaining the Z-disc during contraction by interacting with other proteins such as myotilin and α-actinin via its PDZ domain 21, 25, 44. Studies on mice lacking Cypher have shown disorganized Z-discs and a decrease in sarcomere diameter 9, 26, 27, implying possible deficiencies in sarcomere assembly. Emerging evidence suggests that the sarcomere might be the master regulator of CM maturation 45. Depletion of MYH6 or TPM1 (components of thick filaments) and mutation of ACTN2 (Z-discs protein) have been found to disrupt the organization of T-tubules and the distribution of mitochondrial 10, 17. This indicates that an abnormal sarcomere is sufficient to impair other aspects of CM maturation. In this study, we further validated Cypher as a sarcomeric protein involved in CM maturation. Cypher potentially acts as a scaffold for the biogenesis of T-tubules and mitochondria, and functions as a signal transduction hub for MRTFA-SRF signalling, which affects electrophysiology and oxidative metabolism. Interestingly, ENH (another PDZ-LIM protein) mutant and Cypher-ENH double mutant CMs have reduced sarcomere diameters 46, 47, indicating that ENH may also play a role in mediating CM maturation. Thus, sarcomeres serve not only as crucial cytoskeletal components, but also fulfill a signalling function in CM maturation.

DCM is a leading cause of heart failure; however, its pathophysiology remains ambiguous due to its intricate etiology 24, 48. Recent research has indicated that faulty maturation of CMs may play a role in the development of cardiomyopathy, as the absence of certain proteins related to DCM, including PGC, MYH6, TPM1, RYR2, and SIRT1, can hinder the maturation processes of CMs, such as sarcomere expansion, electrophysiology, and oxidative metabolism 5, 7, 8, 17. However, evidence supporting this hypothesis is limited due to the early occurrence of embryonic lethality and cardiac dysfunction, which obscures cell-autonomous changes in CM maturation. In our study, CypherL-KO mice might serve as an ideal model for studying this relationship. At an earlier stage (P21), CypherL-KO hearts exhibited reduction in sarcomere length and width, a smaller cross-sectional area of mitochondrial, noticeable dilatation of T-tubules, and wider intercalated discs (Figure 1). Analysis of RNA-seq revealed dysregulation of crucial genes associated with oxidative metabolism, sarcomere structure, action potential, and intracellular calcium handling in P3 CypherL-KO hearts (Figure 3). Comparable abnormalities in ultrastructure and gene expression were observed in CypherL-KO mice at 2, 6, and 12 months (Supplementary Figure S2 and Figure 3). The persistent presence of these abnormalities might render the CMs dysfunctional and increase their susceptibility to adverse changes. This is corroborated by our previous research, which demonstrated that young CypherL-KO mice exhibited inotropic and lusitropic dysfunction prior to the onset of DCM, and displayed more pronounced left ventricular dilation and impaired cardiac function subsequent to transverse aortic constriction surgery 27. These findings imply a connection between maturation abnormalities and the susceptibility to DCM.

In our study, we observed that CypherL-KO mice injected with AAV-SRF at postnatal day 1 displayed improved contractility and reduced ventricular dilatation at both 6 and 12 months (Figure 5E-H). Conversely, mice with AAV-SRF injection at postnatal one month exhibited severe DCM at 6 months, as evidenced by significantly reduced EF and FS, and increased LVIDs and ESV (Supplementary Figure S4). These findings suggest that re-expression of SRF during the critical period within the first three weeks after birth can effectively restore cardiac function. Furthermore, the observed CM maturation defects in P21 CypherL-KO mice, including diminished cell area, smaller sarcomere, and reduced expression of mature sarcomere isoforms (Myh6 and Tnni3), were ameliorated upon SRF re-expression (Figure 5A-D). These data provide additional evidence for the relationship between CM maturation and the development of DCM. Nevertheless, more studies are required to validate this association and delve deeper into the underlying mechanisms.

The transcription factor SRF plays a crucial role in the maturation of cardiac myocytes by activating genes that are essential for the assembly of sarcomeres and mitochondrial metabolism 10, 17. In noncardiomyocytes, the transcriptional activity of SRF is regulated by actin dynamics through its cofactor MRTFA 15. In our study, Cypher interacts with actin filaments and inhibits the depolymerisation of F-actin, as observed in skeletal muscle cells 49. Additionally, Cypher promotes the formation of bundles of F-actin without affecting actin polymerisation (Figure 7H-K). Comparable F-actin bundling capabilities have been observed in various sarcomeric proteins such as cofilin-1, α-actinin-2, myotilin and profilin 16, 18, 19. Moreover, Cypher modulates actin-mediated MRTFA nuclear localisation, thereby promoting SRF signalling during CM maturation. Similar mechanisms have been described in CM maturation defects caused by α-actinin mutation 10. Treatment with Latrunculin B (an actin polymerisation inhibitor) and CCG-1423 (an MRTFA inhibitor) resulted in a comparable immature phenotype and decreased expression of maturation-related proteins, similar to the effects observed in Cypher knockdown NRCMs (Supplementary Figure S6). Our experimental data support the hypothesis that actin-mediated MRTFA-SRF signalling serves as a connection between sarcomere and CM maturation. Meanwhile, cytoskeletal proteins such as lamin and myopalladin have also been reported to affect MRTFA-SRF signalling by modulating actin dynamics 14, 15, suggesting their potential involvement in CM maturation. While MRTFA nuclear translocation typically enhances SRF activity without affecting its expression 50, our study revealed notable alterations in SRF protein levels, likely due to autoregulation whereby SRF activation induces the expression of genes, including SRF itself 14, 15. Interestingly, we observed a mutual influence between SRF and MRTFA. In Cypher knockdown NRCMs, the decreased MRTFA protein levels were significantly elevated after SRF re-expression (Supplementary Figure S5A, B). As expected, MRTFA inhibitor treatment led to a reduction in MRTFA expression, which was also accompanied by a decrease in SRF expression (Supplementary Figure S6A, B). The synergistic role of MRTFA and SRF in the heart remains an intriguing topic that warrants further investigation.

The maturation of CMs derived from pluripotent stem cells (PSC) is crucial for their effective use in regenerative medicine 1, 3, 35. Current studies have indicated that mechanical cues, matrix elasticity, substrate stiffness, static and dynamic stretching, play an essential role in the morphological and functional maturation of CMs 51, 52. However, the mechanisms by which CMs sense and convert mechanical forces into molecular signals during the maturation process are still not fully understood. The actin-mediated shuttling of MRTFA is widely acknowledged as a pivotal intermediary in the mechanotransduction pathways 53. Our findings have revealed that the signalling cascade involving actin-mediated MRTFA-SRF can effectively modulate the maturation of CMs, thereby offering valuable insights into the mechanisms underlying incomplete maturation *in vitro* culture and facilitating the advancement of maturation strategies for PSC-CMs.

In conclusion, sarcomeric protein Cypher/ZASP promotes CM maturation through the actin-MRTFA-SRF signalling pathway. SRF supplement during the critical perinatal period restores CM maturation and notably prevents the progression of DCM in mice with Cypher deletion. These findings establish a connection between impairments in neonatal CM maturation and the susceptibility of cardiomyopathy and heart failure.

### Study approval

All experimental procedures conform to the NIH guidelines and were approved by the Animal Ethics Committee of the First Affiliated Hospital, Zhejiang University School of Medicine, Hangzhou, China (approval no.2018273-1).

## Supplementary Material

Supplementary figures and tables.

## Abbreviations

AAV-SRF: Adeno-associated virus encoding SRF
AAV-EGFP: Adeno-associated virus encoding enhanced green fluorescent protein
Ad-SRF: Adenoviruses encoding serum response factor
Ad-GFP: Adenoviruses encoding green fluorescent protein
CM: Cardiomyocyte
CO-IP: Co-immunoprecipitation
CypherL: Cypher long isoforms
DAPI: 4′,6-diamidino-2-phenylindole
DCM: Dilated cardiomyopathy
DEGs: Differentially expressed genes
EDV: End-diastolic volume
EF: Ejection fraction
ESV: End-systolic volume
FS: Fractional shortening
GAPDH: glyceraldehyde-3-phosphate dehydrogenase
GO: Gene Ontology
GSEA: Gene set enrichment analysis
HW/BW: Heart to body weight ratio
ICDs: Intercalated discs (ICDs)
KEGG: Kyoto Encyclopedia of Genes and Genomes
LVIDs: Left ventricle internal dimension at systole
LVPWs: Left ventricular posterior wall thickness at systole
MRTFA: Myocardin-related transcription factor A
NRCMs: Neonatal rat cardiomyocytes
P21: Postnatal day 21
PSC: Pluripotent stem cells
RT-qPCR: Real-time quantitative polymerase chain reaction
SiCypher: Small interfering RNA targeting Cypher
SiScramble: Scrambled small interfering RNA
SRF: Serum response factor
TEM: Transmission electron microscopy
WGA: Wheat germ agglutinin
ZASP: Z-band alternatively spliced PDZ-motif protein

## References

[1] Guo Y, Pu WT (2020). Cardiomyocyte Maturation: New Phase in Development. Circ Res.

[2] Maroli G, Braun T (2021). The long and winding road of cardiomyocyte maturation. Cardiovasc Res.

[3] Karbassi E, Fenix A, Marchiano S, Muraoka N, Nakamura K, Yang X (2020). Cardiomyocyte maturation: advances in knowledge and implications for regenerative medicine. Nat Rev Cardiol.

[4] VanDusen NJ, Lee JY, Gu W, Butler CE, Sethi I, Zheng Y (2021). Massively parallel in vivo CRISPR screening identifies RNF20/40 as epigenetic regulators of cardiomyocyte maturation. Nat Commun.

[5] Fang Y, Fan W, Xu X, Janoshazi AK, Fargo DC, Li X (2022). SIRT1 regulates cardiomyocyte alignment during maturation. J Cell Sci.

[6] Zhang H, Pei L, Ouyang Z, Wang H, Chen X, Jiang K (2022). AP-1 activation mediates postnatal cardiomyocyte maturation. Cardiovasc Res.

[7] Guo Y, Cao Y, Jardin BD, Zhang X, Zhou P, Guatimosim S (2022). Ryanodine receptor 2 (RYR2) dysfunction activates the unfolded protein response and perturbs cardiomyocyte maturation. Cardiovasc Res.

[8] Murphy SA, Miyamoto M, Kervadec A, Kannan S, Tampakakis E, Kambhampati S (2021). PGC1/PPAR drive cardiomyocyte maturation at single cell level via YAP1 and SF3B2. Nat Commun.

[9] Peter AK, Cheng H, Ross RS, Knowlton KU, Chen J (2011). The costamere bridges sarcomeres to the sarcolemma in striated muscle. Prog Pediatr Cardiol.

[10] Guo Y, Cao Y, Jardin BD, Sethi I, Ma Q, Moghadaszadeh B (2021). Sarcomeres regulate murine cardiomyocyte maturation through MRTF-SRF signaling. Proc Natl Acad Sci U S A.

[11] Toepfer CN, Garfinkel AC, Venturini G, Wakimoto H, Repetti G, Alamo L (2020). Myosin Sequestration Regulates Sarcomere Function, Cardiomyocyte Energetics, and Metabolism, Informing the Pathogenesis of Hypertrophic Cardiomyopathy. Circulation.

[12] Colpan M, Iwanski J, Gregorio CC (2021). CAP2 is a regulator of actin pointed end dynamics and myofibrillogenesis in cardiac muscle. Commun Biol.

[13] Yotti R, Seidman CE, Seidman JG (2019). Advances in the Genetic Basis and Pathogenesis of Sarcomere Cardiomyopathies. Annu Rev Genomics Hum Genet.

[14] Ho CY, Jaalouk DE, Vartiainen MK, Lammerding J (2013). Lamin A/C and emerin regulate MKL1-SRF activity by modulating actin dynamics. Nature.

[15] Filomena MC, Yamamoto DL, Caremani M, Kadarla VK, Mastrototaro G, Serio S (2020). Myopalladin promotes muscle growth through modulation of the serum response factor pathway. J Cachexia Sarcopenia Muscle.

[16] Le Dour C, Chatzifrangkeskou M, Macquart C, Magiera MM, Peccate C, Jouve C (2022). Actin-microtubule cytoskeletal interplay mediated by MRTF-A/SRF signaling promotes dilated cardiomyopathy caused by LMNA mutations. Nat Commun.

[17] Guo Y, Jardin BD, Zhou P, Sethi I, Akerberg BN, Toepfer CN (2018). Hierarchical and stage-specific regulation of murine cardiomyocyte maturation by serum response factor. Nat Commun.

[18] Kostan J, Pavsic M, Puz V, Schwarz TC, Drepper F, Molt S (2021). Molecular basis of F-actin regulation and sarcomere assembly via myotilin. PLoS Biol.

[19] Sun H, Qiao Z, Chua KP, Tursic A, Liu X, Gao YG (2018). Profilin Negatively Regulates Formin-Mediated Actin Assembly to Modulate PAMP-Triggered Plant Immunity. Curr Biol.

[20] Zheng M, Cheng H, Banerjee I, Chen J (2010). ALP/Enigma PDZ-LIM domain proteins in the heart. J Mol Cell Biol.

[21] Zhou Q, Ruiz-Lozano P, Martone ME, Chen J (1999). Cypher, a striated muscle-restricted PDZ and LIM domain-containing protein, binds to alpha-actinin-2 and protein kinase C. J Biol Chem.

[22] Xuan T, Wang D, Lv J, Pan Z, Fang J, Xiang Y (2020). Downregulation of Cypher induces apoptosis in cardiomyocytes via Akt/p38 MAPK signaling pathway. Int J Med Sci.

[23] Huang C, Zhou Q, Liang P, Hollander MS, Sheikh F, Li X (2003). Characterization and in vivo functional analysis of splice variants of cypher. J Biol Chem.

[24] Schultheiss HP, Fairweather D, Caforio ALP, Escher F, Hershberger RE, Lipshultz SE (2019). Dilated cardiomyopathy. Nat Rev Dis Primers.

[25] Zhou Q, Chu PH, Huang C, Cheng CF, Martone ME, Knoll G (2001). Ablation of Cypher, a PDZ-LIM domain Z-line protein, causes a severe form of congenital myopathy. J Cell Biol.

[26] Zheng M, Cheng H, Li X, Zhang J, Cui L, Ouyang K (2009). Cardiac-specific ablation of Cypher leads to a severe form of dilated cardiomyopathy with premature death. Hum Mol Genet.

[27] Cheng H, Zheng M, Peter AK, Kimura K, Li X, Ouyang K (2011). Selective deletion of long but not short Cypher isoforms leads to late-onset dilated cardiomyopathy. Hum Mol Genet.

[28] Vatta M, Mohapatra B, Jimenez S, Sanchez X, Faulkner G, Perles Z (2003). Mutations in Cypher/ZASP in patients with dilated cardiomyopathy and left ventricular non-compaction. J Am Coll Cardiol.

[29] Wang DF, Lyu JL, Fang J, Chen J, Chen WW, Huang JQ (2019). Impact of LDB3 gene polymorphisms on clinical presentation and implantable cardioverter defibrillator (ICD) implantation in Chinese patients with idiopathic dilated cardiomyopathy. J Zhejiang Univ Sci B.

[30] Pathak P, Blech-Hermoni Y, Subedi K, Mpamugo J, Obeng-Nyarko C, Ohman R (2021). Myopathy associated LDB3 mutation causes Z-disc disassembly and protein aggregation through PKCalpha and TSC2-mTOR downregulation. Commun Biol.

[31] Huang W, Zhu PJ, Zhang S, Zhou H, Stoica L, Galiano M (2013). mTORC2 controls actin polymerization required for consolidation of long-term memory. Nat Neurosci.

[32] Kannan S, Kwon C (2020). Regulation of cardiomyocyte maturation during critical perinatal window. J Physiol.

[33] Vermij SH, Abriel H, van Veen TA (2017). Refining the molecular organization of the cardiac intercalated disc. Cardiovasc Res.

[34] Wheeler MA, Warley A, Roberts RG, Ehler E, Ellis JA (2010). Identification of an emerin-beta-catenin complex in the heart important for intercalated disc architecture and beta-catenin localisation. Cell Mol Life Sci.

[35] Sakamoto T, Kelly DP (2023). Cardiac maturation. J Mol Cell Cardiol.

[36] Wang Y, Yao F, Wang L, Li Z, Ren Z, Li D (2020). Single-cell analysis of murine fibroblasts identifies neonatal to adult switching that regulates cardiomyocyte maturation. Nat Commun.

[37] Ang YS, Rivas RN, Ribeiro AJS, Srivas R, Rivera J, Stone NR (2016). Disease Model of GATA4 Mutation Reveals Transcription Factor Cooperativity in Human Cardiogenesis. Cell.

[38] Giacomelli E, Meraviglia V, Campostrini G, Cochrane A, Cao X, van Helden RWJ (2020). Human-iPSC-Derived Cardiac Stromal Cells Enhance Maturation in 3D Cardiac Microtissues and Reveal Non-cardiomyocyte Contributions to Heart Disease. Cell Stem Cell.

[39] Sahli Costabal F, Choy JS, Sack KL, Guccione JM, Kassab GS, Kuhl E (2019). Multiscale characterization of heart failure. Acta Biomater.

[40] Ge J, Burnier L, Adamopoulou M, Kwa MQ, Schaks M, Rottner K (2018). RhoA, Rac1, and Cdc42 differentially regulate alphaSMA and collagen I expression in mesenchymal stem cells. J Biol Chem.

[41] Parreno J, Raju S, Wu PH, Kandel RA (2017). MRTF-A signaling regulates the acquisition of the contractile phenotype in dedifferentiated chondrocytes. Matrix Biol.

[42] McElhinny AS, Schwach C, Valichnac M, Mount-Patrick S, Gregorio CC (2005). Nebulin regulates the assembly and lengths of the thin filaments in striated muscle. J Cell Biol.

[43] Lin C, Guo X, Lange S, Liu J, Ouyang K, Yin X (2013). Cypher/ZASP is a novel A-kinase anchoring protein. J Biol Chem.

[44] Lv J, Pan Z, Chen J, Xu R, Wang D, Huang J (2021). Phosphoproteomic Analysis Reveals Downstream PKA Effectors of AKAP Cypher/ZASP in the Pathogenesis of Dilated Cardiomyopathy. Front Cardiovasc Med.

[45] Ahmed RE, Tokuyama T, Anzai T, Chanthra N, Uosaki H (2022). Sarcomere maturation: function acquisition, molecular mechanism, and interplay with other organelles. Philos Trans R Soc Lond B Biol Sci.

[46] Cheng H, Kimura K, Peter AK, Cui L, Ouyang K, Shen T (2010). Loss of enigma homolog protein results in dilated cardiomyopathy. Circ Res.

[47] Mu Y, Jing R, Peter AK, Lange S, Lin L, Zhang J (2015). Cypher and Enigma homolog protein are essential for cardiac development and embryonic survival. J Am Heart Assoc.

[48] Wang D, Fang J, Lv J, Pan Z, Yin X, Cheng H (2019). Novel polymorphisms in PDLIM3 and PDLIM5 gene encoding Z-line proteins increase risk of idiopathic dilated cardiomyopathy. J Cell Mol Med.

[49] Lin X, Ruiz J, Bajraktari I, Ohman R, Banerjee S, Gribble K (2014). Z-disc-associated, alternatively spliced, PDZ motif-containing protein (ZASP) mutations in the actin-binding domain cause disruption of skeletal muscle actin filaments in myofibrillar myopathy. J Biol Chem.

[50] Vartiainen MK, Guettler S, Larijani B, Treisman R (2007). Nuclear actin regulates dynamic subcellular localization and activity of the SRF cofactor MAL. Science.

[51] Rashid SA, Blanchard AT, Combs JD, Fernandez N, Dong Y, Cho HC (2022). DNA Tension Probes Show that Cardiomyocyte Maturation Is Sensitive to the Piconewton Traction Forces Transmitted by Integrins. ACS Nano.

[52] Tallawi M, Rai R, Boccaccini AR, Aifantis KE (2015). Effect of substrate mechanics on cardiomyocyte maturation and growth. Tissue Eng Part B Rev.

[53] Osmanagic-Myers S, Kiss A, Manakanatas C, Hamza O, Sedlmayer F, Szabo PL (2019). Endothelial progerin expression causes cardiovascular pathology through an impaired mechanoresponse. J Clin Invest.

