# Supplementary material for: Cypher/ZASP drives cardiomyocyte maturation via actin-mediated MRTFA-SRF signalling
Source: Theranostics. 2024 Jul 22;14(11):4462–80. doi: 10.7150/thno.98734 (PMC11303069; doi:10.7150/thno.98734)
Supplement: Supplementary file 1 — Supplementary figures and tables. [file thnov14p4462s1.pdf]

Supplementary figures

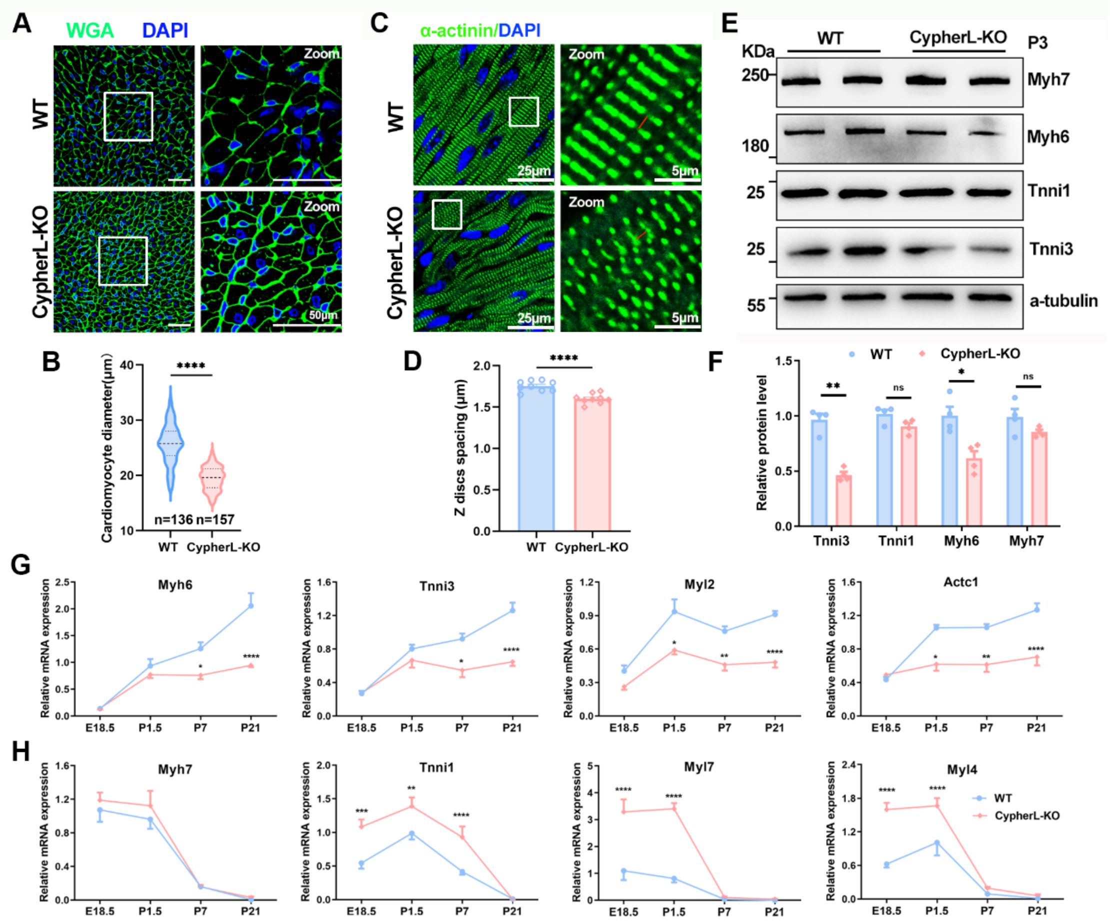

Figure S1. Effect of Cypher on cardiomyocyte (CM) maturation. (A) WGA staining in cardiac tissue sections from P21 WT and CypherL-KO mice. DAPI was used to label nuclei. Scale bars: 50 µm. **(B)** Statistical results of CM diameters (n = 4). **(C–D)** Immunostaining of α-actinin in P21 WT and CypherL-KO mice. DAPI labels the nuclei. The distance between Z-discs is highlighted using red lines, statistical results (D, n = 3). Scale bars: 25 or 5 µm. **(E–F)** Western blot analysis of immature (Myh7 and Tnni1) and mature (Myh6 and Tnni3) myofibrillar isoforms in WT and CypherL-KO hearts at P3 with α-tubulin as a loading control, n = 4. **(G–H)** Real-time quantitative polymerase chain reaction (RT-qPCR) analysis of mature (G) and immature (H) myofibrillar isoforms in WT and CypherL-KO hearts from E18.5 to P21, n = 4–5, with GAPDH as a reference gene. Data represent the mean ± SEM. Unpaired two-tailed Student's t test (B, D, F) and two-way repeated-measures ANOVA followed by Bonferroni's post hoc test (G, H), *$p < 0.05$; **$p < 0.01$; ***$p < 0.001$; ****$p < 0.0001$; ns, no significant difference.

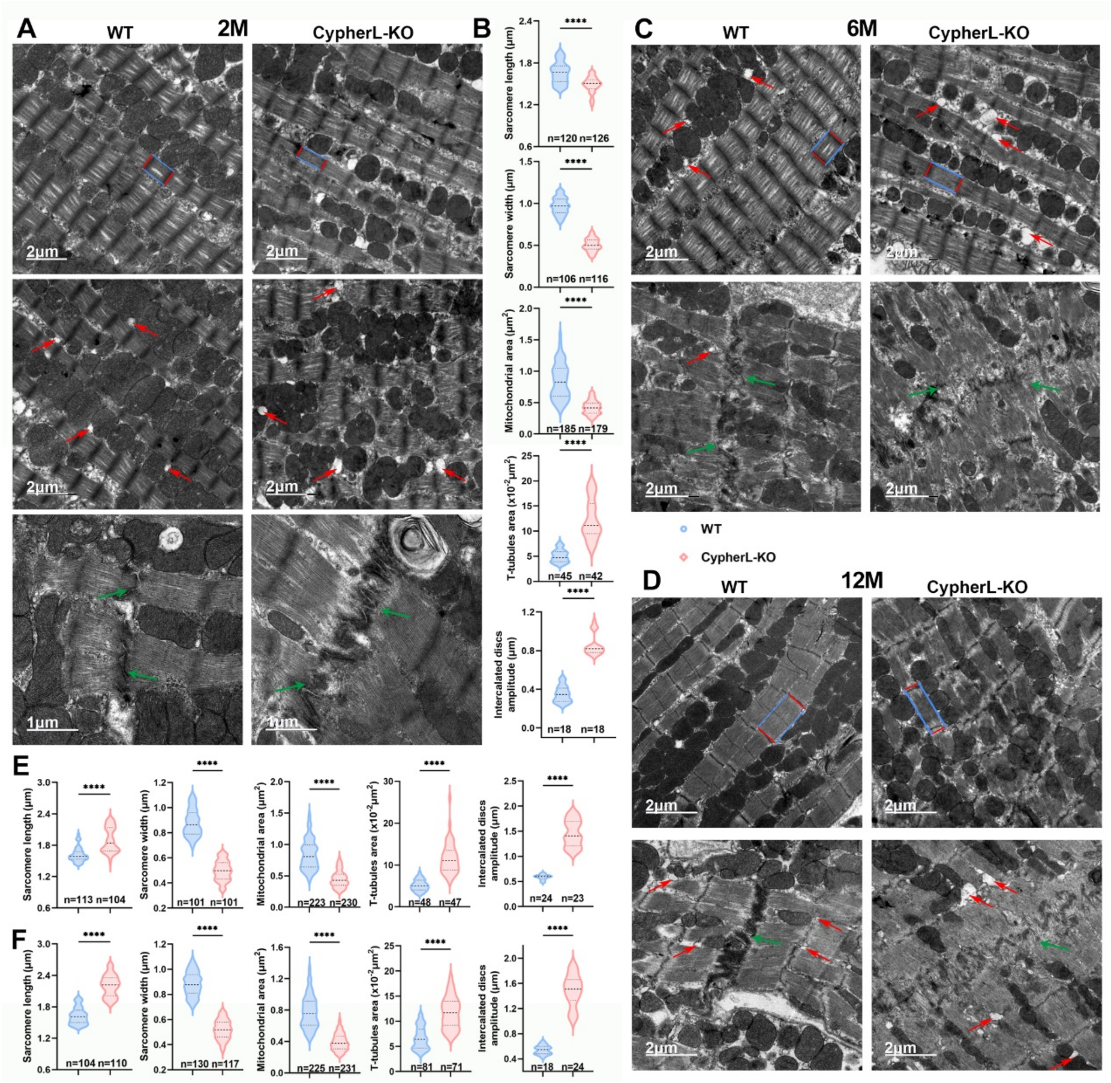

Figure S2. Ultrastructural disorders related to CM maturation in the progression of dilated cardiomyopathy caused by Cypher deletion. Electron micrographs showing the ultrastructural characteristics of CMs in WT and CypherL-KO mice at the age of 2 months (A, B), 6 months (C, E) and 12 months (D, F). Sarcomere length and width are highlighted using blue or red lines. T-tubules and intercalated discs are indicated by red and green arrows, respectively. Scale bars: 2 or 1 μm. Quantitative analysis (n = 3). Data represent the mean ± SEM. Unpaired two-tailed Student's t test (B, E, F), ****$p < 0.0001$.

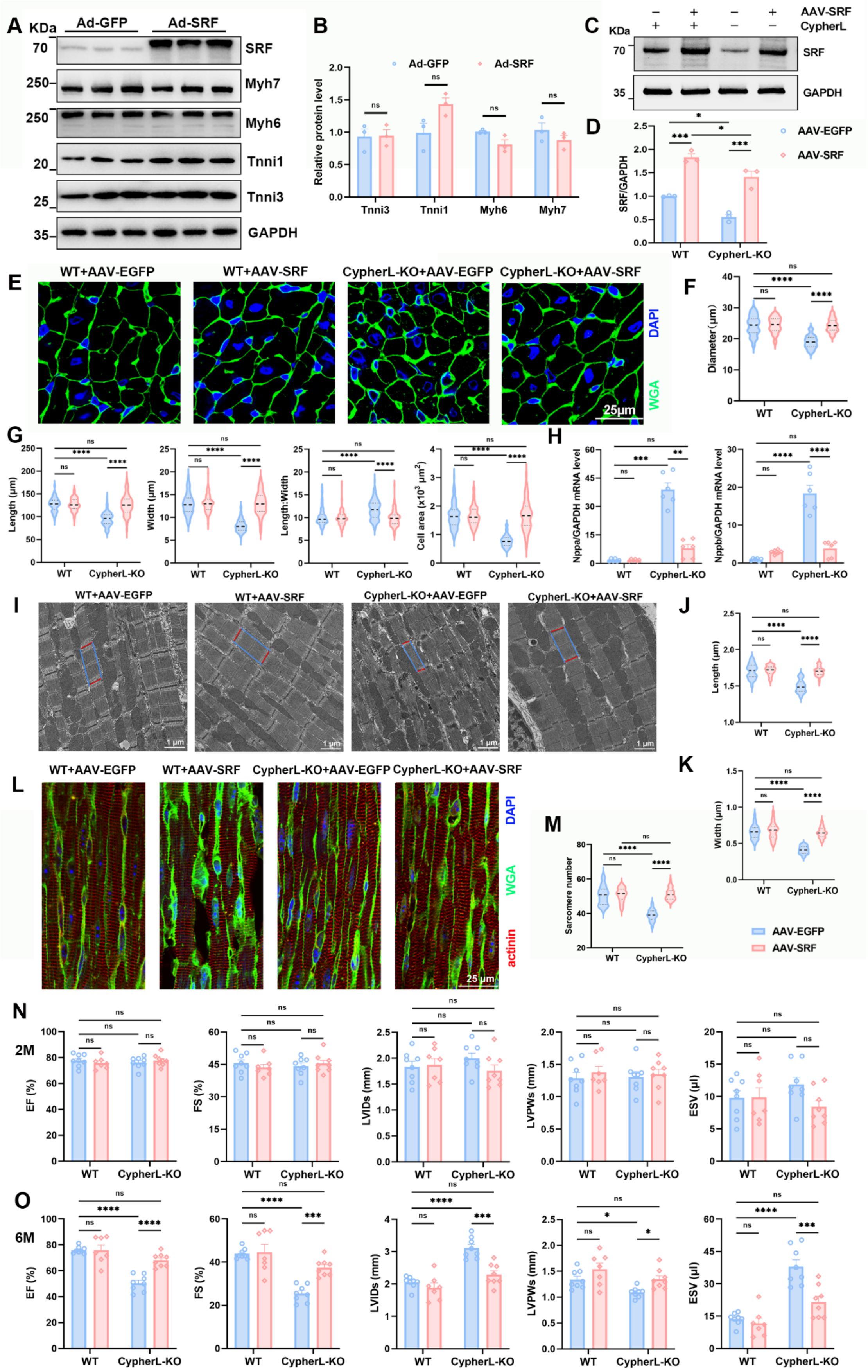

A
KDa
Ad-GFP
Ad-SRF
SRF
Myh7
Myh6
Tnni1
Tnni3
GAPDH
B
Ad-GFP
Ad-SRF
Relative protein level
Tnni3
Tnni1
Myh6
Myh7
C
AAV-SRF
CypherL
D
AAV-EGFP
AAV-SRF
SRF/GAPDH
WT
CypherL-KO
E
WT+AAV-EGFP
WT+AAV-SRF
CypherL-KO+AAV-EGFP
CypherL-KO+AAV-SRF
DAPI
WGA
25μm
F
Diameter (μm)
G
Length (μm)
Width (μm)
Length:Width
Cell area (x10³ μm²)
H
Nppa/GAPDH mRNA level
Nppb/GAPDH mRNA level
I
J
K
L
actinin
25 μm
M
Sarcomere number
N
2M
EF (%)
FS (%)
LVIDs (mm)
LVPWs (mm)
ESV (μl)
O
6M

Figure S3. Effect of SRF re-expression on the maturation of cardiomyocytes and the cardiac function of mice. (A–B) Western blot analysis of SRF, immature (Myh7 and Tnni1), and mature (Myh6 and Tnni3) myofibrillar isoforms in neonatal NRCMs infected with control adenovirus (Ad-GFP) or adenovirus carrying SRF (Ad-SRF), GAPDH was probed as a loading control. Statistical results in (B, $n = 3$). Similar results were observed in 3 independent experiments. **(C–D)** The protein levels of SRF in WT and CypherL-KO hearts at 21 days of age after adeno-associated virus encoding SRF (AAV-SRF) or control AAV-EGFP injection. GAPDH was used as the internal reference. Quantifications are shown in D ($n = 3$). **(E)** WGA staining in cardiac tissue sections from P21 WT and CypherL-KO mice after AAV-SRF or AAV-EGFP injection. DAPI was used to label nuclei. Scale bars: 50 µm. **(F)** Statistical results of CM diameters ($n = 3$). **(G)** Quantification of length, width, length-to-width ratio, and cell area of CMs in P21 WT and CypherL-KO mice infected with AAV-SRF or AAV-EGFP ($n = 3$). **(H)** RT-qPCR analysis of cardiac foetal genes (Nppa and Nppb) in P21 WT and CypherL-KO mice infected with AAV-SRF or control AAV-EGFP ($n = 6$), with GAPDH as a reference gene. **(I)** Electron micrographs of sarcomere structures in P21 WT and CypherL-KO hearts injected with AAV-SRF or control AAV-EGFP. Sarcomere length and width are highlighted using blue or red lines. Scale bars: 1 µm. **(J–K)** Statistical analysis ($n = 3$). **(L)** WGA and actinin co-staining of cardiac tissue sections from P21 WT and CypherL-KO mice injected with AAV-SRF or control AAV-EGFP. Scale bars: 25 µm. **(M)** Quantitative analysis of sarcomere numbers within each cardiomyocyte ($n = 3$). **(N–O)** Cardiac function in 2- and 6-month-old WT and CypherL-KO mice injected with AAV-EGFP or AAV-SRF. Echocardiographic parameters in 2-month-old mice (N, $n = 7–8$) and 6-month-old mice (O, $n = 7–8$). EF, ejection fraction; FS, fractional shortening; LVIDs, left ventricle internal dimension at systole; LVPWs, left ventricular posterior wall thickness at systole; ESV, end-systolic volume. Data represent the mean ± SEM. Unpaired two-tailed Student's *t* test (B) and two-way ANOVA followed by Bonferroni's post hoc test (D–O), $*p < 0.05$; $**p < 0.01$; $***p < 0.001$; $****p < 0.0001$; ns, no significant difference.

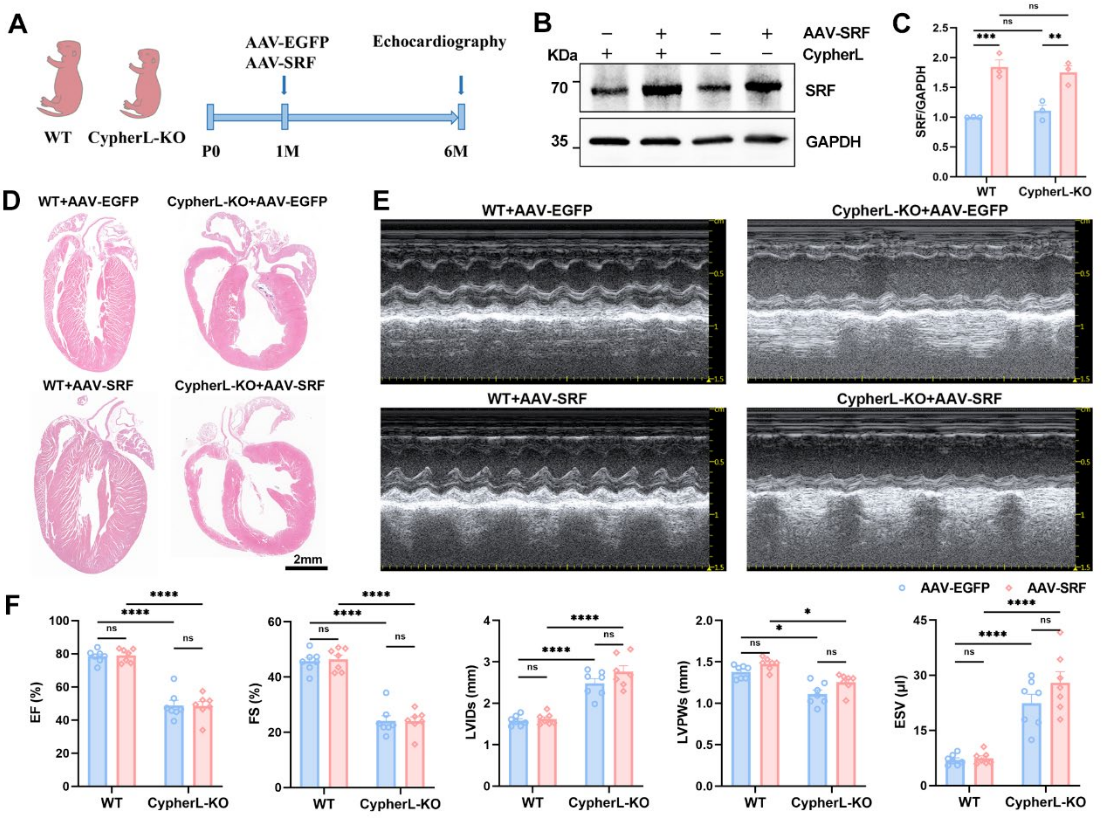

Figure S4. The re-expression of SRF from postnatal one month did not exhibit any ameliorative effect on cardiac function in CypherL-KO mice. (A) Schematic diagram of the experimental strategy. One-month-old WT and CypherL-KO mice were injected subcutaneously with AAV-SRF or control AAV-EGFP, and sacrificed after echocardiography detection at the age of 6 months. **(B–C)** The protein level of SRF in WT and CypherL-KO hearts at 21 days after AAV-EGFP or AAV-SRF injection. GAPDH was used as the internal reference (n = 3). **(D)** Representative H&E images of cardiac tissue sections from 6-month-old WT and CypherL-KO mice infected with AAV-SRF or control AAV-EGFP. Scale bars: 2 mm. **(E)** Representative echocardiographs of 6-month-old WT and CypherL-KO mice injected with AAV-SRF or control AAV-EGFP. **(F)** Statistical analysis of echocardiographic parameters. EF, ejection fraction; FS, fractional shortening; LVIDs, left ventricle internal dimension at systole; LVPWs, left ventricular posterior wall thickness at systole; ESV, end-systolic volume. (n = 7). Data represent the mean ± SEM. Two-way ANOVA followed by Bonferroni's post hoc test (C, F), *$p < 0.05$; **$p < 0.01$; ***$p < 0.001$; ****$p < 0.0001$; ns, no significant difference.

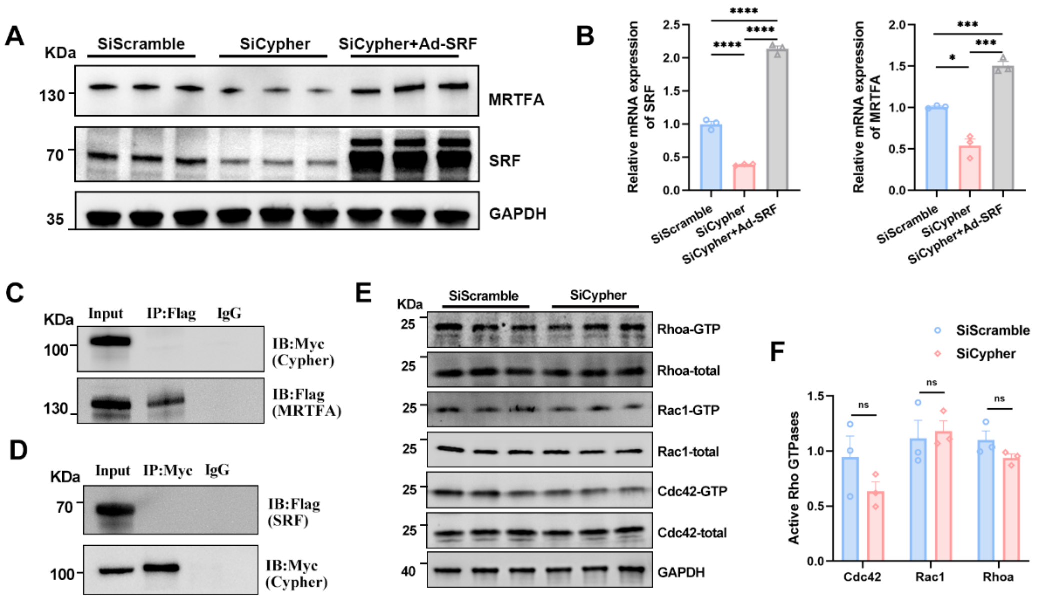

Figure S5. Cypher modulates the SRF-MRTFA pathway without direct interaction or affecting Rho GTPase activity. (A–B) Western blots of SRF and MRTFA in NRCMs treated with SiScramble, SiCypher, and SiCypher upon Ad-SRF infection, with GAPDH as a loading control. Quantitative analysis (n = 3). **(C–D)** Co-immunoprecipitation of Cypher and SRF or MRTFA in 293T cells. Myc-tagged Cypher and Flag-tagged MRTFA (C) or SRF (D) were co-transfected to 293T cells. Immunoprecipitation was performed using anti-Flag (C) or anti-Myc (D) antibody, and the samples were further probed with anti-Myc or anti-Flag antibody. IP, immunoprecipitation; IB, immunoblotting. Mouse IgG served as the negative control. **(E–F)** Protein levels of Rho GTPases (Rhoa, Rac1, Cdc42) and their active GTP-bound forms in NRCMs treated with SiScramble or SiCypher, GAPDH was used as a loading control. Statistical results in F (n = 3). Similar results were obtained from 3 independent cellular experiments. Data represent the mean ± SEM. Unpaired two-tailed Student's t test (F) and one-way ANOVA with Tukey's post hoc test (B), *p < 0.05; ***p < 0.001; ****p < 0.0001; ns, no significant difference.

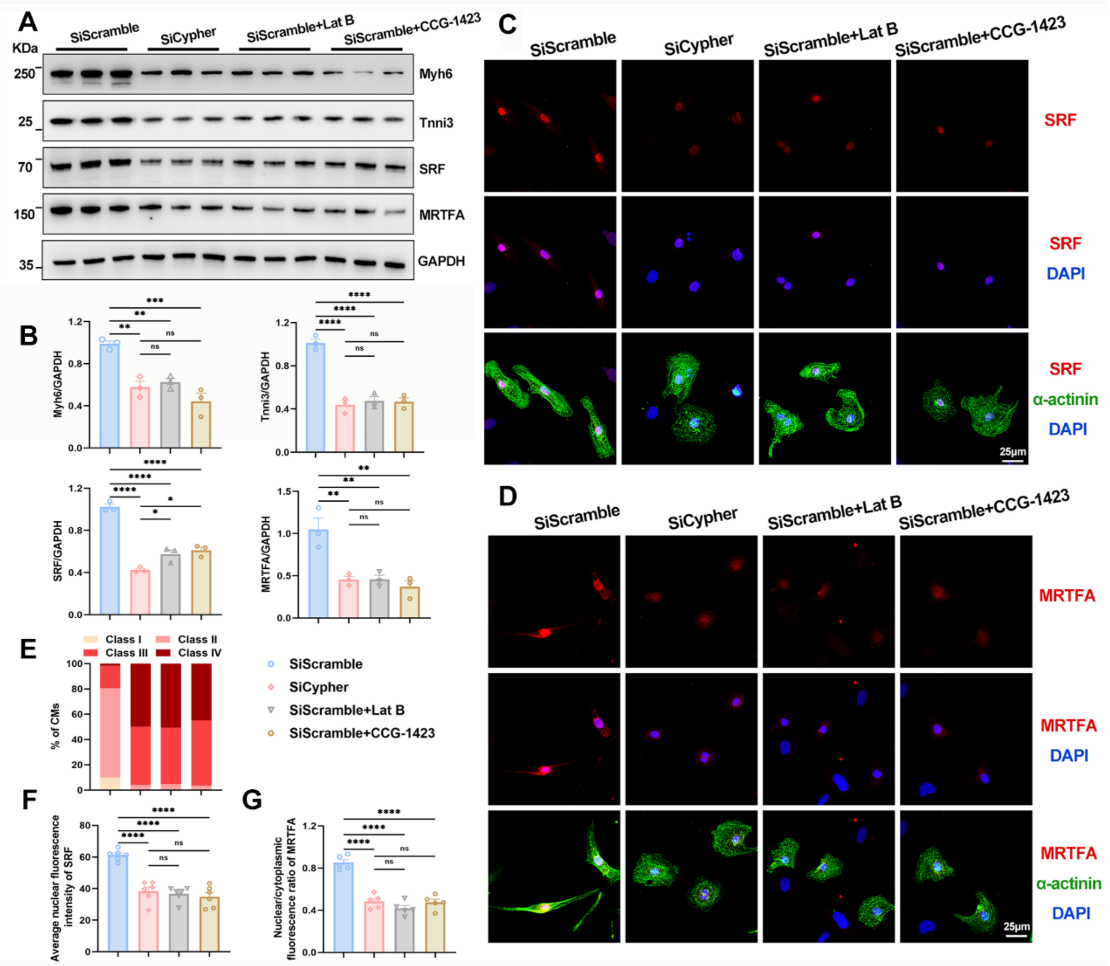

Figure S6. The inhibition of actin-MRTFA-SRF signalling causes CM maturation defects in NRCMs. **(A–B)** Protein levels of SRF, MRTFA and mature (Myh6 and Tnni3) myofibrillar isoforms in NRCMs transfected with SiScramble (control) or small interfering RNA against Cypher (SiCypher) for 48 h, or treated with latrunculin B (actin polymerisation inhibitor, 0.1 μM) and CCG-1423 (MRTFA inhibitor, 10 μM) for 24 h. GAPDH was used as the internal reference. (B) Pooled data (n = 3). **(C–D)** Immunostaining of SRF (C) and MRTFA (D) in NRCMs treated with SiScramble, SiCypher, latrunculin B (Lat B, 0.1 μM, 24 h), and CCG-1423 (10 μM, 24 h) labelled with DAPI (nuclei) and α-actinin (CMs). Scale bars: 25 μm. **(E)** Morphological classification of NRCMs treated with SiScramble (n = 485), SiCypher (n = 475), latrunculin B (0.1 μM, 24 h; n = 433), and CCG-1423 (10 μM, 24 h; n = 401). **(F)** SRF nuclear fluorescence intensity, 6 randomly selected fields of view were quantified for each group. **(G)** Nuclear to cytoplasmic fluorescence ratio of MRTFA, 5 randomly selected fields of view were quantified for each group. Data represent the mean ± SEM. Similar results were obtained from 3 independent cellular experiments. One-way ANOVA with Tukey's post hoc test (B, F, and G), *$p < 0.05$; **$p < 0.01$; ***$p < 0.001$; ****$p < 0.0001$; ns, no significant difference.

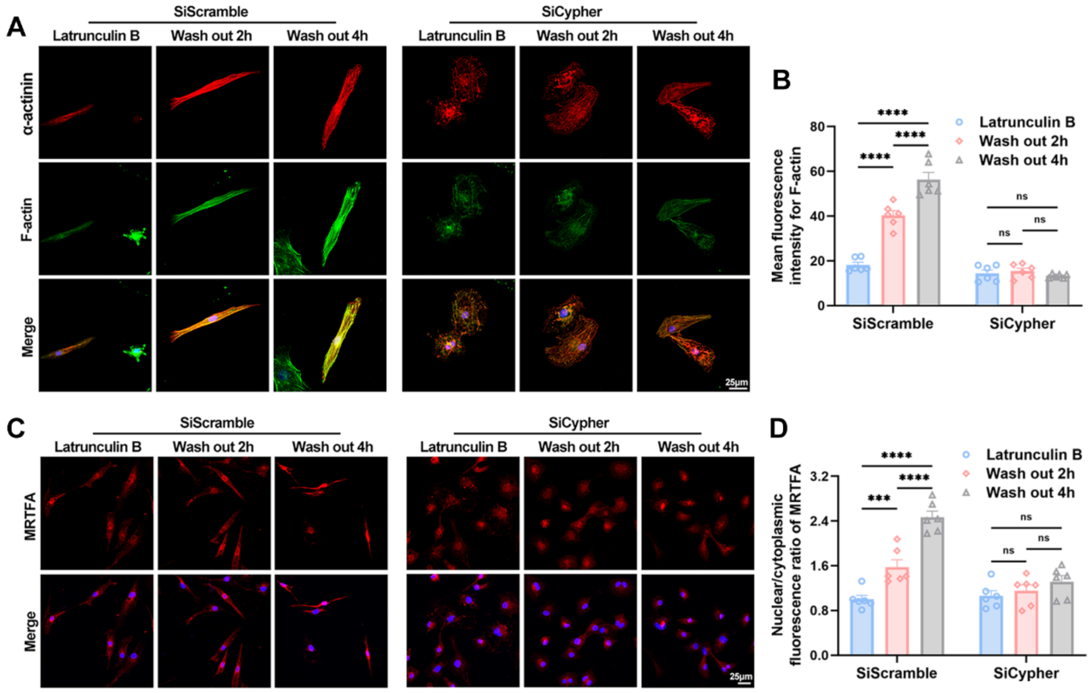

Figure S7. Cypher deficiency disturbs actin dynamics and MRTFA nuclear localisation. (A–B) Immunofluorescence images of SiScramble- or SiCypher-transfected NRCMs after latrunculin B washout. NRCMs were treated with 10 μM latrunculin B for 1 h, followed by 2 or 4 h drug washout. F-actin and cardiomyocytes were labelled with phalloidin and α-actinin, respectively. Scale bars: 25 μm. F-actin fluorescence intensity (B), 6 randomly selected fields of view were quantified for each group. **(C–D)** Immunostaining for MRTFA in NRCMs after latrunculin B washout. Nuclei were labelled DAPI. Scale bars: 25 μm. Nuclear to cytoplasmic MRTFA ratio (D), 6 randomly selected fields of view were quantified for each group. Data represent the mean ± SEM. Similar results were obtained from 3 independent cellular experiments. Two-way ANOVA followed by Bonferroni's post hoc test (B and D), ***$p < 0.001$; ****$p < 0.0001$; ns, no significant difference.

Supplementary tables

Table S1. List of primer sequences for RT-qPCR.

Gene	Species	Sequences (5'-3')
Myh7	Mouse	Forward: ACTGTCAACACTAAGAGGGTCA
		Reverse: TTGGATGATTTGATCTTCCAGGG
Tnni1	Mouse	Forward: ATGCCGGAAGTTGAGAGGAAA
		Reverse: TCCGAGAGGTAACGCACCTT
Myl7	Mouse	Forward: GGCACAACGTGGCTCTTCTAA
		Reverse: TGCAGATGATCCCATCCCTGT
Myl4	Mouse	Forward: AAGAAACCCGAGCCTAAGAAGG
		Reverse: TGGGTCAAAGGCAGAGTCCT
Myh6	Mouse	Forward: GCCCAGTACCTCCGAAAGTC
		Reverse: GCCTTAACATACTCCTCCTTGTC
Tnni3	Mouse	Forward: TCTGCCAACTACCGAGCCTAT
		Reverse: CTCTTCTGCCTCTCGTTCCAT
Myl2	Mouse	Forward: ATCGACAAGAATGACCTAAGGGA
		Reverse: ATTTTTCACGTTCACTCGTCCT
Actc1	Mouse	Forward: CTGGATTCTGGCGATGGTGTA
		Reverse: CGGACAATTTCACGTTCAGCA
SRF	Mouse	Forward: GGCCGCGTGAAGATCAAGAT
		Reverse: CACATGGCCTGTCTCACTGG
LDB3	Mouse	Forward: AAGAAGCCCTTTGGGAACA
		Reverse: CAGACCGCACAAATGAAGC
Nppa	Mouse	Forward: GCTTCCAGGCCATATTGGAG
		Reverse: GGGGGCATGACCTCATCTT
Nppb	Mouse	Forward: GAGGTCACTCCTATCCTCTGG
		Reverse: GCCATTTCCTCCGACTTTTCTC
GAPDH	Mouse	Forward: AGGTCGGTGTGAACGGATTTG
		Reverse: TGTAGACCATGTAGTTGAGGTCA
Myh7	Rat	Forward: GCAAAGGCAAAGCAAAGAAA
		Reverse: GCAGCGTACAAAGTGAGGGT
Tnni1	Rat	Forward: AGGTGGGAGACTGGAGGAAG
		Reverse: GAGGGAACAACAACAGCAAA
Acta2	Rat	Forward: GGGCATCCACGAAACCACCTA
		Reverse: GCCGCCGATCCAGACAGAATA
Myl7	Rat	Forward: GGAGGGTAAGTGTCCCAGAGG
		Reverse: GGGTCAAACATTCGGAAAGCA
Myh6	Rat	Forward: ATGCTGGCACCGTGGACTA
		Reverse: GAGTTTGAGGGAGGACTTCTGG
Tnni3	Rat	Forward: AGATTGCGAAGCAGGAGATGGAG
		Reverse: AAACTTGCCACGCAGGTCATAGA
Acta1	Rat	Forward: GCCTCACTTCCTACCCTCG
		Reverse: TCAGGATACCTCGCTTGCT
Myl2	Rat	Forward: AAGGCAAAGGGTCGCTGAA

		Reverse: TCTCCGTGGGTGATGATGTG
SRF	Rat	Forward: GTTACACGACCTTCAGCAAGA
		Reverse: CAAAGCCAGTGGCACTCAT
LDB3	Rat	Forward: TGACCAGGCAGCAAGGAAC
		Reverse: GACTCCATCAATGGCCACCA
GAPDH	Rat	Forward: AGTGCCAGCCTCGTCTCATA
		Reverse: GATGGTGATGGGTTTCCCGT

Table S2. List of antibodies.

Antigen （Application）	Cat.#	Working concentration	Source
WGA-488 (IF)	W11261	1:500	Invitrogen
α-tubulin (WB)	Ab7291	1:5000	Abcam
Tnni3 (WB/IF)	Ab209809	1:2000/1:200	Abcam
Tnni1 (WB)	Ab203515	1:2000	Abcam
Myh6 (WB)	Ab50967	1:1000	Abcam
Myh7 (WB)	22280-1-AP	1:1000	Proteintech
β-catenin (IF)	Ab32572	1:200	Abcam
α-actinin (IF)	Ab9465	1:200	Abcam
Cypher (WB/IF)	H00011155-M06	1:2000/1:200	Abnova
SRF (WB)	Ab252868	1:1000	Abcam
SRF (IF)	16821-1-AP	1:100	Proteintech
GAPDH-HRP (WB)	HRP-60004	1:10000	Proteintech
α-actin (WB)	0407-3	1:1000	HuaBio
Phalloidin-488 (IF)	Ab176753	1:800	Abcam
MRTFA (IF)	21166-1-AP	1:100	Proteintech
MRTFA (WB)	A8504	1:500	Abclonal
Histone H3 (WB)	Ab201456	1:2000	Abcam
Myc (IP/WB)	2276	1:100/1:1000	CST
Flag (IP/WB)	AF519	1:100/1:1000	Beyotime
Cdc42 (WB)	21010	1:500	NewEast
RhoA (WB)	21017	1:500	NewEast
Rac1 (WB)	21003	1:500	NewEast
